# Mechanisms of Silver Nanoparticle Release, Transformation and Toxicity: A Critical Review of Current Knowledge and Recommendations for Future Studies and Applications

**DOI:** 10.3390/ma6062295

**Published:** 2013-06-05

**Authors:** Bogumiła Reidy, Andrea Haase, Andreas Luch, Kenneth A. Dawson, Iseult Lynch

**Affiliations:** 1 Centre for BioNano Interactions, School of Chemistry and Chemical Biology, University College Dublin, Belfield, Dublin 4, Ireland; E-Mails: kenneth.a.dawson@cbni.ucd.ie (K.A.D.); iseult.lynch@cbni.ucd.ie (I.L.); 2 Department of Product Safety, German Federal Institute for Risk Assessment (BfR), Berlin 10589, Germany; E-Mails: Andrea.Haase@bfr.bund.de (A.H.); andreas.luch@bfr.bund.de (A.L.); 3 Department of Geography, Earth and Environmental Sciences, University of Birmingham, Edgbaston, Birmingham B15 2TT, UK

**Keywords:** physico-chemical characterisation, interaction with environmental components, dissolution, agglomeration, biological impacts, coatings, cytotoxicity

## Abstract

Nanosilver, due to its small particle size and enormous specific surface area, facilitates more rapid dissolution of ions than the equivalent bulk material; potentially leading to increased toxicity of nanosilver. This, coupled with their capacity to adsorb biomolecules and interact with biological receptors can mean that nanoparticles can reach sub-cellular locations leading to potentially higher localized concentrations of ions once those particles start to dissolve or degrade *in situ*. Further complicating the story is the capacity for nanoparticles to generate reactive oxygen species, and to interact with, and potentially disturb the functioning of biomolecules such as proteins, enzymes and DNA. The fact that the nanoparticle size, shape, surface coating and a host of other factors contribute to these interactions, and that the particles themselves are evolving or ageing leads to further complications in terms of elucidating mechanisms of interaction and modes of action for silver nanoparticles, in contrast to dissolved silver species. This review aims to provide a critical assessment of the current understanding of silver nanoparticle toxicity, as well as to provide a set of pointers and guidelines for experimental design of future studies to assess the environmental and biological impacts of silver nanoparticles. In particular; in future we require a detailed description of the nanoparticles; their synthesis route and stabilisation mechanisms; their coating; and evolution and ageing under the exposure conditions of the assay. This would allow for comparison of data from different particles; different environmental or biological systems; and structure-activity or structure-property relationships to emerge as the basis for predictive toxicology. On the basis of currently available data; such comparisons or predictions are difficult; as the characterisation and time-resolved data is not available; and a full understanding of silver nanoparticle dissolution and ageing under different conditions is observed. Clear concerns are emerging regarding the overuse of nanosilver and the potential for bacterial resistance to develop. A significant conclusion includes the need for a risk—benefit analysis for all applications and eventually restrictions of the uses where a clear benefit cannot be demonstrated.

## 1. Introduction 

Nanosilver, in the form of colloidal silver, has been used for more than 150 years and has been registered as a biocidal material in the United States since 1954 [1]. There is anecdotal evidence of its use as far back as ancient Egypt and Rome. Silver can form many different inorganic and organic complexes, and its most stable oxidation states are +0 and +1, although others (+2, +3) exist as well.

Silver nitrate is often used as a precursor in the synthesis of the different forms of silver nanoparticles. Nanosilver exists in a huge variety of different forms. Silver nanoparticles can be of different shape (spheres, rods, wires, triangles, ...), coatings (citrate, polymer, peptide, sugars, ...) and of different sizes (from few up to 100s nm). In the following sections we present some of the applications of silver nanomaterials and discuss how their physical properties influence their chemical and biological properties. We also provide insight into current knowledge gaps and give pointers for experimental design to ensure comparability of data.

## 2. Silver Nanoparticles in Use—Routes of Exposure 

Nanosilver, in terms of product number, is one of the nanomaterials with the highest degree of commercialization, as roughly 30% of all products currently registered in nano-product databases claim to contain nanosilver [2]. However, recent estimates indicate that the mass of Ag-NP produced is very low compared to other NPs [3]. It actually compares well with the mass of (ionic) silver that has been found in the atmosphere for weather control purposes about 30 years ago [4], since silver levels were much higher during peak photographic times as recorded by the silver content in sewage sludge.

Furthermore, in a variety of emerging materials, silver nanoparticles are often combined with other substances to develop combined functionalities.

Nanosilver in the form of colloidal silver has been used for more than 100 years, and has been registered as a biocidal material in the United States since 1954 [1]. So-called “colloidal silver”, which contains silver of different concentrations and particle sizes, has historically mainly been used for treatment of wounds and infections but with the invention of modern antibiotics in the 1940s its use was largely reduced. Since the 1990s it is turning back to the markets mainly as an alternative medicine treatment. However, there is no evidence of the beneficial effects of “colloidal silver treatment” in alternative medicine, and the U.S. National Center for Complementary and Alternative Medicine states that marketing claims made about “colloidal silver” are scientifically unsupported [5,6]. Silver—containing products aimed to be used as a mineral supplement were found to be toxic and their administration is not recommended [7]. Given this long history of usage, readers are referred to a good discussion of all the different silver forms that are used (not just the nano-Ag applications) which helps to place nano-silver in the correct context [8]. Here however, we focus specifically on nano-forms of silver, and describe some of the most prevalent application areas below.

Since any risk is the product of exposure times hazard, an estimate of likely exposure concentrations to Ag nanoparticles is vital. However, the concentration and form of nanomaterials in the environment are difficult to quantify and methodological progress is needed, although sophisticated exposure models show that predicted environmental concentrations (PECs) for Ag NPs in different environmental compartments are at the range of ng L^−1^ to mg kg^−1^ [9]. On the other hand, exposure to silver nanoparticles may be more common than we thought and may have been occurring for centuries: Glover *et al.* showed that under certain conditions, metallic silver will release large numbers of silver nanoparticles [10]. Liu *et al.* [11] suggested that ionic silver might be the form that is used to transport nanosilver within the body and out of which nanosilver can be reformed in body compartments.

### 2.1. Textiles

Textiles containing nano-silver (and other forms of silver) make up the majority of commercially available nano-functionalized materials [12]. In the exposure model developed by Blaser *et al.* [13], silver-functionalized textiles and plastic are considered as one of the main sources of silver in the environment. Silver is used in T-shirts, socks, underwear, sports clothing and many others [14]. There are different ways to produce silver functionalized textiles. Silver (or other nanoparticles) may be embedded into the fibres or applied to the surface of the fibres. The preparation method will affect the durability of the functionalization and the potential for release of silver nanoparticle or ion into the environment [15]. The main concern here is the ease with which these nanoparticles can enter the water system via washing effluent, and whether that can have a negative impact on the bacterial colonies in waste water treatment plants (see Section 3 for further details).

### 2.2. Food Packaging

According to Cushen [16] silver nanomaterials are used in the food industry and research. There are several examples of food packaging containing silver nanoparticles, which are or were commercially available (Table 1).

**Table 1:** Silver nanoparticle use in the food industry ([16], modified).

Product	Function of nanocomponent	Commercial status	Further information	Reference
BlueMoonGoods_ Fresh Box Silver Nanoparticle Food Storage Containers	Antimicrobial	Withdrawn from website	Nanoparticles permanently embedded in the container	[17]
Nano Care Technology, Ltd. Antibacterial Kitchenware	Antimicrobial	URL no longer available	–	[18]
Sunriver Industrial nanosilver fresh food bag	Antimicrobial	Commercially available	Ag has been shown to migrate from these bags	[19]
FresherLonger_ Plastic Storage Bags	Antimicrobial	Commercially available but antimicrobial and Ag nanoparticles have been removed from the description	Resealable zip lock	[18]

It is important to notice that only a few forms of silver are assessed by EFSA for use in food contact materials and thus only those are allowed in food packaging materials [20,21]. Nanoscaled silver is currently not assessed by EFSA and thus it is not approved for food contact materials sold in Europe (note however that the regulation is different for different types of food contact materials).

### 2.3. Implants and Other Medical Devices 

Due to its antimicrobial properties, the use of silver in wound dressings, dental hygiene, and treatment of eye conditions and other infections is well established [6,22]. Silver compounds can be used in much lower concentrations, and also in applications such as plastics where high temperature processing is required that is not feasible for organic compounds which would be degraded at such temperatures [1]. Materials containing silver are used to produce surgical meshes (for bridging large wounds and as reinforcements to tissue repair). Other uses of silver-enriched materials include vascular prosthesis, ventricular drainage catheters and the orthopedics. It was shown that the overall rate of catheter-related blood stream infections was significantly lower when silver impregnated central venous catheters (CVC) than in the conventional format [5]. Future therapeutic directions may include nano-silver use as an anti-inflammatory and as anti-platelet agents and antiviral drugs [5]. Surface modification of implants using silver does not affect their biocompatibility while resulting in an added benefit due to increased antibacterial properties [22]. Many devices are still at the stage of development, while others, like silver-impregnated catheters, are already available for clinical use. The plasmonic properties of silver nanoparticles make them a good material to use in other medical devices (e.g., biosensors) [23].

Acticoat^®^ is the first commercial wound dressing made up of two layers of polyamide ester membranes covered with nanocrystalline silver. Many studies concerning its antimicrobial properties have been published in recent years [24,25]. Acticoat^®^ reduced the frequency of burn wound sepsis and secondary bacteremia. Silver in the form of nanoparticles seems to promote healing and achieve better cosmetic results (compared with the other silver compounds tested). The proposed mechanism is that silver nanoparticles facilitate the proliferation and migration of keratinocytes, reduce the formation of collagen by fibroblasts and modulate the number of cytokines produced [5].

Importantly, nanosilver is a very effective fungicide [26] as well as having antiviral properties [27]. In the study of Wright *et al.* [26], the nanocrystalline silver-based dressing Acticoat^®^ Antimicrobial Barrier dressing was confirmed to provide the fastest and broadest-spectrum fungicidal activity among all tested wound dressings (including those containing silver nitrate or silver sulfadiazine). It also overcomes several problems associated with previously used wound-dressings, like tissue irritation and insufficiently wide spectrum of antifungal properties.

The antiviral properties of small (5–20 nm) human serum albumin stabilized silver nanoparticles (synthetized in HEPES buffer) were investigated by Sun *et al. [27]*. The studied nanoparticles exhibited a dose-dependent anti-retrovirus activity and inhibited HIV-1 replication. Those antiviral properties, the mechanism of which is not known, did not seem to be dependent on the synthesis method. In comparison, gold nanoparticles did not exhibit strong antiviral properties. The silver nanoparticles used in the study did not show cytotoxic properties towards Hut/CCR5 cells. The antiviral properties of silver nanoparticles have been recently reviewed by Galdiero *et al. [28]*.

### 2.4. Other Consumer Products

The antimicrobial properties of silver make it suitable for use in water treatment processes, surface coatings, including washing machines and paints, and others. Antimicrobial nanomaterials are relatively inert in water and they are not expected to produce harmful disinfection byproducts. Functional nanomaterials, including those containing silver, can be used in high-performance, small-scale or point-of-use water treatment systems. This can be of particular importance in water systems not connected to a central network, and for emergency response following catastrophic events [29]. Nowack *et al.* [1] mention silver-impregnated water filters (where the presence of silver nanoparticles <100 nm was confirmed), which have been used for domestic water purification for decades.

Additionally, different nano-forms of silver can be potentially used in the field of electronics (transparent conducting films, transparent electrodes for flexible devices, flexible thin film tandem solar cells *etc.*) [30]. Here applications exploit their conductivity or electrical properties rather than antimicrobial properties, and exposure/release of nano or ionic silver will be minimal during use but may become an issue upon disposal.

### 2.5. Silver Nanoparticles in Combination with Other Materials

There are several kinds of new materials, whose promising properties (e.g., electronic, antimicrobial) can be used in many fields, but which are not commercialized yet (e.g., silver nanowire networks buried in polymer matrices which can be used as flexible, thermally stable, transparent conductors [30]. The electrical properties of silver nanoparticles can be used also in medical devices. Titanium embedded with silver nanoparticles demonstrates micro-galvanic effects that give rise to both controlled antibacterial activity (inhibited growth of *Staphylococcus aureus* and *Escherichia coli*) and excellent compatibility with osteoblasts (low surface toxicity, enhanced proliferation), which makes this a promising new material for implants [31]. As demonstrated by the authors, the beneficial properties of new materials are related to the micro-galvanic effect between the silver nanoparticles and the titanium matrix. A similar mechanism is proposed for biofilm-inhibiting silver-palladium surfaces that kill bacteria by generating microelectric fields as well as by electrochemical redox processes [32]. The surface of metallic silver has only a slight antimicrobial effect because of its chemical stability, so it is not useful as an antibacterial surface coating. The authors underline the importance of structure—in the new, silver-palladium material the designed distance between the Ag and Pd particles is less than 5 μm to ensure a high local strength of the microelectric fields, as a potential difference over a short distance can give high field strengths (~100 mV/μm). The authors show some evidence that Ag-Pd surfaces can also kill bacteria via redox processes and, in the case of silver-sensitive bacteria, via the release of toxic levels of silver ions from the surface. Despite its evident antimicrobial properties, the designed material did not prevent biofilm formation under all experimental conditions [32].

Several approaches to create antimicrobial nanomaterials have been reported. One is a nanocomposite film, which was proven to have highly efficient antimicrobial properties [33]. Nanocomposite films consist of a commercially available ethylene-vinyl alcohol copolymer (EVOH) embedded with Ag-TiO_2_ nanoparticles. The nanocomposite was proven to exhibit potent antimicrobial activity toward Gram-negative, Gram-positive bacteria/cocci and yeasts, as well as displaying resistance to biofilm formation. Due to the use of silver nanoparticles (and their plasmonic effect) the antimicrobial properties can be enhanced upon irradiation with ultraviolet (UV) light, which opens up the use of visible-light excitation sources. According to the authors, the biocidal action comes from the inorganic-organic interface and takes place on the whole nanocomposite surface. The potential applications include a wide variety of packaging, biomedical coatings and others. Use of Ag-TiO_2_ composites can reduce cost (as titania is widely available and inexpensive) [33].

According to Zhao *et al.* [34] titania nanotubes loaded with silver nanoparticles and fabricated on titanium implants kill all the planktonic bacteria present in a suspension (beef extract-peptone medium at 37 °C) within the first few days. The ability to prevent bacterial adhesion to the implants was maintained without obvious decline for 30 days (long enough to prevent post-operation infection in the early, intermediate and even late stages). Despite some evidence of cytotoxicity, the silver nanoparticle-enriched nanotubes exhibited good tissue integration. The authors concluded that the new material could be used in various applications in orthopedics, dentistry, and other biomedical devices [34], although obtaining approval for such uses may be challenging until more information is known regarding their degradation and/or bioaccumulation profiles.

Relatively inexpensive, non-toxic and biocompatible iron oxide nanoparticles are currently being (or can be potentially) used for many medical applications (e.g., hyperthermia cancer treatments, drug carriers, contrast agents in magnetic resonance imagining (MRI) investigations) [35]. Nanocomposites of iron oxide and silver nanoparticles exhibited very significant antibacterial and antifungal activities against ten tested bacterial strains and four candida species. Minimum inhibition concentrations (MICs) were much lower than the concentrations at which acute toxicity to embryonic mouse fibroblasts was observed [35]. According to the authors, this new nanomaterial could be used for targeted delivery of silver nanoparticles in medicinal and disinfection applications, as, due to the magnetic properties of iron oxide, they can be transported purposely to a certain location for controlled release. However, synthesis and stabilization of non-aggregated magnetic nanoparticles remains challenging, which limits their use. Silica can also be used as a carrier for small silver nanoparticles. Such composites, which are stable at high temperatures, have useful optical properties and can be used in photonic devices [36,37].

## 3. Release of Silver from Functionalized Materials

As products containing silver nanoparticles are widely used (see for example [2], the release of silver nanoparticles (and other forms of silver) into the environment is a potentially serious issue. There are several studies concerning silver release from different materials including textiles [14,15], paints [38] as well as some models predicting the fate of silver in the environment [12,13,39,40]. A key measurement challenge in such studies is to distinguish between nanoparticulate silver and silver ions, as analytical methods such as ICP-MS in its standard mode of operation and elemental analysis can confirm the presence of silver but not distinguish its form (if not preceded by a separation technique, e.g., ultracentrifugation or if not run in single particle mode), whereas methods such as TEM and other particle sizing approaches have difficulty finding low concentrations of particles, and with distinguishing between specific nanoparticles and background particles (especially in products or the environment where there are many other entities of similar size). Combinations of TEM and EDX offer one approach, but are time consuming and costly.

Silver nanoparticles are also used in disinfecting sprays, deodorants and other cosmetics, mainly as an antimicrobial agent [41]. In the study of Lorentz *et al.* [42] the presence of nanoparticles in sprays (four kinds) was investigated. According to the manufacturers, all four sprays contained nanoparticles. The authors consider sprays as especially important, as inhalation is one of the main routes for uptake of nanoparticles into the human body. Besides the presence of engineered nanoparticles, the generation of nanosized droplets was also investigated. Of the four sprays studied, engineered nanoparticles were found only in two of them [42]. This is important information, as, despite the rapidly growing “nano” market, not all products advertised as nano may actually contain nanoparticles (which makes realistic exposure/release predictions even more difficult). Nanosized aerosols were found in three of the tested sprays. One spray (antiperspirant) contained some silver (6.8 ± 0.7 mg silver/kg according to ICP-MS measurements), but no nanoparticulate forms were identified. The difference between ICP-MS and EDX results may indicate that silver was in ionic, not in particulate form, or that the silver concentration was below the detection limit of EDX. Spherical silver nanoparticles (8 ± 6 nm) were found in antimicrobial plant sprays. The total concentration of silver in the product was 9.1 ± 0.1 mg silver/kg, but no nanosized droplets were generated by the product. According to the manufacturer, the spray contained “nano silver” and was supposed to protect plants from bacteria, viruses, mold, fungus and algae.

In the study of Benn and Westerhoff [14] the release of silver from textiles (socks) was investigated. Six types of socks contained up to a maximum of 1360 μg-Ag/g-sock and leached as much as 650 μg of silver into 500 mL of distilled water over 24 h (amount varied between different kinds of socks, which may suggest differences in the manufacturing process). Silver particles from 10 to 500 nm in diameter were present both in textiles (confirmed by SEM) and in wash water. Released silver was both in colloidal and ionic form. The authors provide detailed description of silver release rates and amounts. These results suggest that the amount of released silver strongly depends on the manufacturing process. Experiments conducted using tap water suggest that this strips silver from the sock fabric less than ultrapure water. In tap water, probably due to the presence of salts, the silver solution could not be characterized as nanoparticle or ionic. The authors consider the transformations of silver nanoparticles in the environment as an important factor in their fate in the environmental.

Another study concerning the release of silver from textiles was that by Geranio *et al.* [15]. After studying nine fabrics, the authors concluded that the amount and form of silver released into the environment strongly depends on the way in which the silver is incorporated into a fabric. According to manufacturers, silver was incorporated into fabric in several ways (Table 2). The amount of released silver varies between 1% and 45% of total silver mass, although textiles were washed only once or twice. The authors also investigated the role of pH, surfactants and oxidizing agents and found that in the relevant washing conditions the nanoparticle dissolution rate is rather low. Silver is released mainly as big (>450 nm) particles. The authors underline the problem of insufficient labeling of nano-products (e.g., no information regarding the amount or form of silver contained in the fabrics). In the study of Farkas *et al.* [43] the silver release from a nanosilver producing washing machine was investigated. According to the patent script, ‘silver solution’ released by the machine (after turning on the “nanowash” function) is described as “… a mixture of water and silver ions (Ag+), and refers to a colloidal solution containing silver ions in a nano-particle state suspended in the water …”. The results showed that the machine released silver into its effluent at an average concentration of 11 μg/L. Silver nanoparticles (10 nm according to TEM) were found in the effluent from the washing machine. Cotton textiles washed in the nanosilver producing washing machine contained up to 4.75 μg silver/g of textile (up to 3 washes were performed). Deactivation of the “nanowash” function decreased, but did not stop, the release of silver into water, so such washing machines should be considered as a steady source of silver in the environment. The last observation is of particular importance: the natural freshwater bacterial community showed a significant, dose dependent reduction (60%–80% reduction of abundance) after exposure to “nanowash” effluent. The impact of silver release on bacterial communities in wastewater treatment plants is discussed later in this section. According to the authors’ estimation, if 20% of the households in Norway purchased a nanosilver producing washing machine, the amount of released silver would be as high as 98.5 kg per year.

**Table 2:** Methods of silver incorporation into fabrics ([15], modified).

Method	Silver content (mg/g)
Conventional textile: electrolytically deposited layer of silver (several μm) on fibre	21.6
Plasma-coated fibre with silver nanoparticles (about 100 nm) embedded in polyester matrix	0.39
AgCl (~200 nm) bound to the fibre surface	0.008
AgCl (~200 nm) incorporated in binder on the fibre surface	0.012
Silver nanoparticles bound to the fibre surface	0.029
Silver nanoparticles incorporated into polyester fibre	0.099
Silver nanoparticles incorporated into fibre	0.242
Silver nanoparticles incorporated inside the synthetic fibres (according to manufacturer)	0.003
Nanosized silver incorporated into cotton fibres (according to manufacturer)	2.66

Release of (nano)silver from outdoor paint (containing 1.5 mg Ag/m^2^ of painted surface; average concentration in wet paint—6.2 mg silver nanoparticles/kg) was investigated by Kaegi *et al.* [38]. The experiment was conducted over 372 days, during which 65 runoff events (see Box 2) occurred (36 of which were analysed). The authors found significant leaching of silver nanoparticles (up to 145 μg Ag/L during the initial runoff events). After one year more than 30% (0.5 mg/m^2^) of the initial mass of silver was released into the environment, of which 80% was released during the first eight runoff events. In contrast, only 1% of titania pigments was released during one year. Released silver particles were mostly <15 nm in size, and further analysis revealed that most of the released metallic silver was transformed into less toxic forms such as Ag_2_S. Wider use of silver-containing outdoor paints can thus contribute to a significant increase in the amount of silver released into the environment since most of the silver nanoparticles from the outdoor paint were gone after 1 year. Despite significant leakage of silver from outdoor paints, it is not taken into consideration in many of the existing models of silver fate in the environment [13].

In the study of Mueller and Nowack [44] the quantities of engineered nanomaterials (including nanosilver) releaxsed to the environmnet were modelled from a life-cycle perspective. According to the authors, the estimated PEC/PNEC ratio for nanosilver indicated that no adverse effects in the environment can be expected. Dissolution or transformation of silver nanoparticlers in the environment were not taken into account, so the concentrations of nanosilver in different environmental compartments are probably much lower than the estimated ones.

Gottschalk *et al.* [40] modeled environmental concentrations of silver (and other) nanoparticles for the U.S., Europe and Switzerland. According to the results, risks to aquatic organisms emerging from silver nano-forms cannot be excluded in sewage treatment effluents and in surface waters. The calculated nanosilver concentrations in different environmental compartments were lower than those of some other kinds of nanomaterials, and the calculated concentrations generally reflect the worldwide production volumes. In comparison to the previously mentioned study of Mueller and Nowack [44] the calculated concentration of nanosilver in surface water was much lower, which could be due to sedimentation processes, which were not taken into account in the previous study [44].

**Figure 1:**
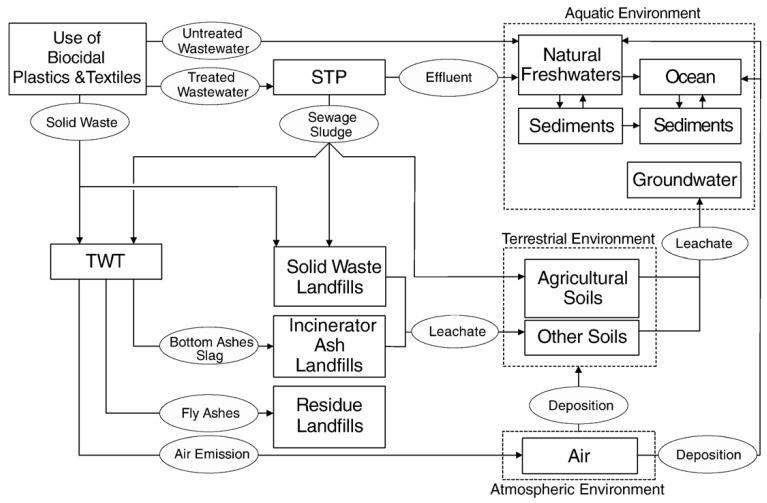
Overview of one model of silver flow triggered by use of biocidal plastics and textiles. Arrows represent silver flows; dashed lines indicate different environmental spheres. TWT = thermal waste treatment; STP = sewage treatment plant [13].

In the study of Blaser *et al.* [13] the risk to freshwater ecosystems emerging from silver incorporated into textiles and plastics and subsequently released into the environment was investigated. All 25 European Union countries were taken into consideration in predicting the usage of silver-enriched materials/products and silver release into the environment. The authors proposed a model of silver mass flow (Figure 1), estimated emission, assessed the fate of silver in a river system, estimated the predicted environmental concentrations (PEC), and evaluated available toxicity data for environmentally relevant forms of silver in order to make an estimation of predicted no-effect concentrations (PNEC) and risk characterisation. According to the authors, silver incorporated into plastics and textiles accounts for up to 15% of total silver released into water systems in the EU (of which nanosilver is only a fraction). Three different emission scenarios were studied, in which silver release was in the range 110–230 t/yr. The authors made an assumption that only silver ion is released into the environment (no whole particles), which is contrary to some available experimental data [14,15]. Marine ecosystems were not considered at all. Emissions from production processes, solid waste from silver containing products and deposition from atmosphere to surface water (5% of amount released to air) were neglected. The study can be used only as a prediction of the fate of a fraction of silver (no sources of silver other than textiles and plastics were taken into account due to unknown or a hard to estimate scales of use in other products). As the recent data confirmed significant silver release from other sources [38,43], there is a need for more general models of silver fate in the environment.

Based on available data, Blaser *et al.* [13] did not predict any risk from dissolved silver originating from plastics and textiles for microbial communities in sewage treatment plants, although concentrations in freshwater ecosystems (particularly in sediments) may exceed safe limits (PEC/PNEC ratio > 1, see Box 1 for definitions). As the majority of silver released into water is incorporated into sewage sludge (which is in agreement with more recent experimental data [45]) and then deposited on landfills or used as a fertiliser, soil and groundwater contamination should also be taken into account. The impact of silver nanoparticles on soil organisms was investigated by Shoults-Wilson *et al.* [46]. Emission to air is negligible (1% of silver leaving sewage treatment plants), as during the incineration process most of silver remains in the ashes. This last statement contradicts Scheringer [47], who claim that the behaviour of nanoparticles in incinerators is largely unknown. Prediction of the removal in wastewater treatment plants does not take into consideration different treatment stages, and total wastewater is not distributed to the correct treatment level (*i.e.*, no treatment, pre-treatment, and primary treatment, secondary treatment), by total mass fraction [39]. According to Benn and Westerhoff [14], the high silver concentration may limit the disposal of the biosolids as an agricultural fertilizer, unless cost effective methods to remove silver can be developed. It is worth also noting that in several studies on silver release, metallic nano-Ag is not detected in significant quantities, and indeed AgCl is the major form of silver found in washing solution by Lorenz *et al.*, suggesting that nano-Ag may not be as widespread as often expected in wastewater and even for human exposure (skin contact to textiles), AgCl may be more important than nano-Ag [48].

According to the life-cycle analysis proposed by Walser *et al.* [12], the release of potentially toxic substances from washed and disposed nanomaterial–enriched textiles is of minor relevance. In the proposed model of the environmental fate of T-shirts containing silver nanoparticles, the authors are more concerned about the fact that according to their prediction, the production of textiles containing nano-silver has an increased carbon footprint compared to the production of conventional textiles. The environmental pollution due to silver mining (if high amounts of silver are required) is also considered as a potential risk.

End of life cycle aspects are also relevant for other nanosilver—containing products, such as consumer products, medical devices *etc.*, especially those released to landfills [49]. Thus, while significant work has been undertaken to understand the environmental fate (release and transformation) of silver, the multiple forms and potential transformations, diverse (sometimes just claimed) applications and conflicting data make a complete assessment of likely exposure concentrations diffucult at present.

Importantly, in existing models the chemical transformation of metallic silver to other silver componennts (discussed in Chapter 6) is usually not taken into account. As transformation processes strongly impact silver toxicity, adopting toxicity data from experiments with metallic silver nanoparticles to models in which silver nanoparticles pass different environmental compartments (beeing thus chemically transformed) is questionable.

**Box 1:**

PEC—Predicted Environmental Concentration, the concentration of the substance which will eventually be found in the environment;
PNEC—Predicted No Effect Concentration, the concentration of the substance below which adverse effects in the environmental compartment of concern are not expected to occur;
PEC/PNEC ratio—an indicator of risk.
All above according to [50]
PEC/PNEC < 1 = No immediate concern
PEC/PNEC = 1–10 = Of concern if supply volumes increase
PEC/PNEC = 10–100 = Further data required
PEC/PNEC > 100 = Reduce risk immediately
(According to Denehurst Chemical Safety Ltd. [51])
Note: PEC/PNEC is sometimes called Risk Characterisation Ratio “RCR”
Runoff—surface flow of water e.g., down the façade [52];
Effluent—the waste liquid from domestic sewage, industrial sites or from agricultural processes. Effluents are harmful when they enter the environment, especially in freshwater, because of their polluting chemical composition;
Pore waters—the water occupying the spaces between sediment particles (U.S. Environmental Protection Agency, 2001, cited from [53]);
Anoxic—total deprivation of oxygen (U.S. Environmental Protection Agency, 2009, cited from [54]);
EC50—median effective concentration: concentration at which 50% of the population are effected in whatever end-point is being assessed [50];
IC50—concentration causing 50% inhibition of a given parameter, e.g., growth [50].

## 4. Behaviour of Silver Nanoparticles in Wastewater Treatment Plants

There are several studies in which experiments or proposed models concerning the behaviour of silver nanoparticles in wastewater treatment plants are presented [39,45].

In the study of Kaegi *et al.* [45] the behaviour of silver nanoparticles in a pilot wastewater treatment plant (WWTP) was investigated. A silver nanoparticle suspension (silver nanoparticles very polydipersed regarding size) was spiked into the influent at a concentration of 2400 μg/L (first 24 h) and 130 μg/L (for following 24 days). After the end of silver addition, the experiment was continued for a further 17 days. On the 11th day of the experiment the excess sludge removal rate was increased from 1 m^3^/day to 2.2 m^3^/day (there was no excess sludge removal during the first 24 h). Freely dispersed nanoparticles were observed in the effluent only during the initial pulse spike. During that time, 70% of the silver left the WWTP with the effluent, but afterwards that fraction decreased to 2%–3% and remained roughly constant until the end of the experiment. After the initial pulse, all silver nanoparticles leaving the WWTP (in effluent or with excess sludge) were associated with biosolids. According to mass balance, ~5% (7.2 g) of the added silver nanoparticles left the WWTP via the effluent, ~85% (110 g) ended up in the excess sludge and ~5% (7.8 g) still remained in the WWTP when the experiment was stopped after 43 days. A better mass balance closure was obtained for the period after the 4th day of experiment (90% in excess sludge, 2.5% in the effluent, 7% remained in WWTP). The authors consider the physical and chemical transformations of silver nanoparticles as crucial for their fate in the environment. The results of the another, batch experiment suggest that most of the metallic silver was transformed into Ag_2_S during the first 2 h. The issue of silver transformation in the environment and its implications is discussed later in this review article (Section 7).

Adams and Kramer [55] measured silver, inorganic sulphide, and thiol compounds in municipal wastewater effluent, receiving waters, and pore waters from an anoxic lake sediment and partitioned the silver into three size fractions: particulate (>0.45 μm), colloidal (10 kDa–0.45 μm), and dissolved (<10 kDa). 30%–35% of the silver in water was in the colloidal form, 15%–20%—in the dissolved form, and most was in particulate form. The dissolved fraction was constant in wastewater treatment plant effluent and receiving waters, which suggests that it is strongly complexed by ligands that are not affected by aggregation or sorption. The particulate silver concentration decreases quickly after reaching the receiving water, probably due to particle settling, sorption (e.g., to soil) and for uptake by organisms. It is important to note that particulate silver in the effluent was not of engineered origin, and indeed there are many reports in the literature of naturally occurring precipitation of silver resulting in formation of nanoparticles, including in alfalfa plants, [56] and in microbes [57] and indeed of engineered silver nanoparticles dissolving and re-precipitating new nanoparticles [10].

Despite the fact that some experimental data is available, nanomaterials’ behaviour in water and wastewater treatment plants remains unclear [39,47]. In the model proposed by O’Brien and Cummins [39] the data available for pharmaceuticals and metals removal (for wastewater treatment plants) and pathogen removal (for water treatment plants) was applied to nanoparticles. The predicted concentrations in surface water were based on the scale of use and were dependent on nanoparticle type. For example, nano-titania concentration, resulting from outdoor paints, was 2 orders of magnitude greater than nano-silver concentration resulting from food packaging (note that this is potentially in contradiction with the study of Kaegi *et al.* [38] where only 1% of titania pigments from outdoor paints was released during one year, however we have not done a detailed comparison or particle loads and conditions to assess this). As mentioned before, O’Brien and Cummins [39] take into account silver-enriched food packaging (Irish market—2 × 10^7^ and 1.2 × 10^6^ kg embedded/bound, respectively). The authors are aware of differences between pharmaceuticals’ and nanoparticles’ behaviour (impact of aggregation and sedimentation, no or low degradation in the case of (most) nanoparticles), so the pharmaceuticals removal model was considered as the “worst-case scenario” [39]. The metal removal model in activated sludge systems includes such processes as physical entrapment of insoluble particles into the floc, active cellular uptake, binding to extra cellular polymers, and volatilization [39] which, according to the results of other studies [45,58], should be relevant also for nanoparticles. As the concentration of natural colloids in freshwater is 2–3 times greater than the suggested nanoparticle release, aggregation and sedimentation are predicted to be the dominant processes in nanoparticle removal. The authors predict also silver (nanoparticles and ions) concentrations at different stages of wastewater and water treatment, using a few scenarios (pharmaceutical/metal removal efficiencies, primary only/secondary treatment) and provide few data series (one for each scenario). Predicted mean annual quantities of silver (nanoparticles and ions) released to surface waters from wastewater treatment plants were 92.5 kg/yr and 68.3 kg/yr (pharmaceutical and metal removal efficiencies, respectively). Predicted mean water concentrations (direct release, sludge leaching, and air deposition included) were 29.5 μg/m^3^ and 21.8 μg/m^3^, respectively. Predicted drinking water concentrations (depending on drinking water scheme used in the model) were 0.9 μg/m^3^ up to 29.5 μg/m^3^ (no water treatment in the last case). Mean nanomaterial removal rates were ~60% (pharmaceutical removal efficiency) and 70% (metal removal efficiency). Based on experimental data, other authors [55] state that the removal of total silver in wastewater treatment was greater than 95%, and still the concentration of silver in the effluent was higher (at least one or two orders of magnitude) than background levels. Based on their findings, the authors do not predict any ecotoxicological or human health risk, although they propose careful monitoring of titania and silver nanoparticle concentrations in surface water.

In the study of O’Brien and Cummins [39] the accumulation of silver (nano and ionic) from food packaging through drinking water was predicted to result in a mean level of 0.37 μg/yr for the general population. The United States Environmental Protection Agency (USEPA) has set water quality criteria values for silver in salt and fresh water at 1.9 and 3.4 μg/L (ppb), respectively.

Another important aspect of silver presence and behaviour in WWTPs is its impact on organisms, which play a crucial role in the biological processes occurring in WWTPs. Blaser *et al.* [13] did not predict any risk for microbial communities in sewage treatment plants. On the other hand, Choi and Hu [59] found that 1 mg/L of small (9–20 nm) silver nanoparticles inhibits growth of nitrifying bacteria by ~80%. Even if such a concentration is not likely to occur under realistic release conditions, further studies concerning this issue are needed, as well as long term impacts studies. A reduction of the abundance of natural freshwater bacteria was also found by Farkas *et al.* [43] in response to the presence of silver nanoparticles.

In the study by Liang *et al.* [60], the impact of silver nanoparticles (average size: 1–29 nm) and silver ions on nitrifying organisms in activated sludge was investigated. According to the authors, silver nanoparticles were more toxic (41.4% nitrification inhibition at concentration 1 mg/L (shock loading), than silver ions (13.5% nitrification inhibition at the same concentration). Nitrification inhibition was observed for more than one month after the silver administration, which indicates that silver nanoparticles can, if concentrations are high enough, cause problems with biological processes in WWTPs. Silver (without distinguishing between ionic and particulate form) was detectable in the sludge for 20 days after the shock loading, most of it complexed with humic acid and other organic acids. Only a small fraction of silver (again without distinguishing between ionic and particulate form) was present in water. An increase of ammonia/nitrite concentration in wastewater effluent was observed. The population of some nitrifying microorganisms (*Nitrospira*) significantly decreased, while others (*Nitrobacter*) were washed away (removed from the WWTP). At the same time, silver nanoparticles did not seem to affect the growth of heterotrophs responsible for organic matter removal.

Based on the available data we can presume that after passing through a WWTP, nanosilver is generally transformed to AgS_2_ (of much lower toxicity), in the same way that dissolved silver is transformed to AgCl. Unintentional uptake by humans cannot be excluded and medical devices and consumer products remain the most likely uptake routes (in most cases controlled and intentional). As pointed out by Johnston *et al.* [61], it is apparent that silver nanoparticles are able to pass through the gastrointestinal tract (GIT), dermal, and lung barriers into the blood, and thereby become distributed throughout the body. It is well established that the tissue distribution of nanoparticles is size dependent [62]. It remains unclear however whether the silver uptake, distribution, and accumulation in the various studies were accounted for by silver nanoparticles and/or ions. Additionally, data is missing on how silver nanoparticle transformation in the environment influences their biological properties.

Thus, an impact of silver nanoparticles on bacterial communities in WWTP and natural waters has been found by many authors [43,59,60]. The wide-spread use of consumer products containing silver may lead to more frequent or general occurrence of bacterial resistance against silver. On the other hand, natural bacterial communities, which we rely upon for wastewater treatment, may suffer due to release of silver from functionalized materials, medical devices *etc.* Those two phenomena should be taken into account while deciding on the permitted use and regulation of silver nanoparticles, as part of the risk-benefit analysis.

## 5. Characterization of Silver Nanomaterials and Its Importance—Lack of Sufficient and Relevant Characterization of Materials Used in Experiments

Despite the fact that there are many studies concerning the impacts of silver nanoparticles on cells or whole organisms, proper analysis and comparison of the available data is very difficult. One of the reasons for this is the lack of sufficient characterization of the nanoparticles used in experiments under the exposure conditions utilized which makes it hard to correlate any observed effect to the nanoparticles’ properties and the available dose (which may be greatly affected by media components). As the morphology and size of silver nanoparticles often differ from the ones reported by manufacturers, their in-house characterization is very important [63].

In most experiments, cells or organisms are exposed to nanoparticles suspended in solution—water, buffer, cell culture medium *etc.* Sufficient characterization of nanoparticles in the same solution and under the same conditions (temperature, time) as used in the experiment seems to be crucial for understanding what actually interacts with cells/organisms and should be performed whenever possible. Often, nanoparticles are characterized in water, but no characterization in culture (exposure) medium is performed [64]. In the study of Foldbjerg *et al.* [63] characterization was performed in medium with lower content of serum (1% instead of 10% as would be typical for *in vitro* studies) and using RPMI instead of DMEM as the medium, despite the use of DMEM + 10% serum in the toxicity studies. Also for *in vivo* studies characterization in often performed in water or buffer rather than in a more relevant medium (e.g., blood plasma in the case of intravenous administration of nanoparticles) [62], although there are some studies where nanoparticles were characterized before and after incubation in the appropriate exposure medium [65,66,67]. In the study of Austin *et al.* [23] the stability of peptide-conjugated particles was investigated after 24 h of incubation in culture medium (10% serum). In the study of Braydich-Stolle *et al.* [68] changes in the physicochemical properties of the nanoparticles were evaluated prior to, during, and after incubation in ALF (artificial lysosomal fluid, simulated biological fluid of pH 4.5). The obtained data lead to the conclusion that the coating (hydrocarbon or polysaccharide) was degraded, in particular after integration into lysosomes, and the particles appear to be decreasing in size with time because of the loss of the coating and silver ions potentially dissociating into solution. Clearly this information is of critical importance in assessing the biological impacts of the nanoparticles and the kinetics of such impacts.

Kittler *et al.* [69] also underline that silver nanoparticle behaviour in biological media is different than in water (e.g., impact of reducing sugars and other biomolecules). Differences between silver nanoparticles behaviour in deionized water and biological medium are also pointed out by Lee *et al.* [70]. O’Brien and Cummins [39] point out that the particles employed in experiments were formulated to avoid agglomeration and so may not be representative of their behaviour in natural ligand containing freshwaters.

Among the multiple nanoparticle physico-chemical characteristics (see for example the OECD list [71,72], some are of particular importance for determining their biological interactions and impacts: size and size dispersion (monodispersity, polydispersity), shape, zeta potential, agglomeration and dissolution rate. Agglomeration (see Box 2 for definition) and dissolution of silver nanoparticles, together with the complexity of silver chemistry in aquatic media, has been already discussed as part of the release sub-section and is covered in further detail in Section 7 on transformation. In this section only size, shape, surface charge and coating will be discussed, and readers are referred to an excellent review of methods for nanoparticle characterization in complex milieu for further details [73].

**Box 2:**

*Agglomerate*: A collection of weakly bound particles or aggregates or mixtures of the two where the resulting external surface area is similar to the sum of the surface areas of the individual components.
**Note 1**: The forces holding an agglomerate together are weak forces, for example “van der Waals” forces, or simple physical entanglement.
**Note 2**: Agglomerates are also termed secondary particles and the original source particles are termed primary particles.
*Aggregate*: A particle comprising of strongly bonded or fused particles where the resulting external surface area may be significantly smaller than the sum of calculated surface areas of the individual components.
**Note 1**: The forces holding an aggregate together are strong forces, for example covalent bonds, or those resulting from sintering or complex physical entanglement.
**Note 2**: Aggregates are also termed secondary particles and the original source particles are termed primary particles.
**Source: ISO TS 27687:2008.** This document can be purchased from the website of the ISO central secrétariat[74]. Copyright remains with ISO. An agreed set of terminology, based on the ISO terminology, was also developed by NanoImpactNet, and is available (free) from [75].

### 5.1. Size

In many studies the impact of silver nanoparticles was found to be size-dependent [61,64,76,77], although this is not a rule [64,78]. In the study of Powers *et al.* [64] counter-size dependent effects of PVP-coated silver nanoparticles were found. In many studies, smaller nanoparticles were found to be most toxic [59] which is often explained by the easier uptake [59] and larger surface area of the same mass of silver [61] which can facilitate faster dissolution and release of silver ions. In most of those studies a mass based dosimetry was used (surface area was not taken into account). In other studies, smaller particle size was not necessarily correlated with more dissolved silver [76,78], suggesting that coatings and interactions with biomolecules can influence dissolution rate, and need to be reported in detail to facilitate interpretation of the data, and indeed factored into the data interpretation. As ionic or biomolecular species in solution can affect the size and size distribution of particles, characterization in the exposure solution is essential.

There are several methods, based on quite different physical principles, which can be used to assess nanoparticle size, with different degrees of effectiveness for more complex samples:

- Light scattering based methods (Dynamic Light Scattering, Nanoparticle Tracking Analysis), in which size is calculated basing on the scattering of light by particles. DLS is widely used to assess the actual hydrodynamic size and nanoparticles behaviour (agglomeration, dissolution) in medium (e.g., [79]). DLS provides also data on nanoparticles size distribution and polydispersity index. DLS suits spherical, not too small particles, best. However it may be difficult or even impossible to analyse heterogeneous mixtures or polydisperse samples, or nanoparticles in the presence of other nanosized entities such as protein clusters. Nanoparticle Tracking Analysis (NTA) allows individual nanoparticles in a suspension to be microscopically visualized (though not, of course, imaged) and their Brownian motion to be separately but simultaneously analysed and from which the particle size distribution (and changes therein) can be obtained on a particle-by-particle basis. This enables separation of particle populations by size and intensity and allows complex and heterogeneous samples to be characterised easier [80].
- Microscopy-based techniques. The most common one is transmission electron microscopy (TEM) [45,59,79,81,82]. The obtained data includes particle and aggregate/agglomerate sizes, shape and potentially crystal structures [79]. Size distribution can be obtained using relevant software, although large numbers of particles must be counted to get statistically relevant data, which is even more difficult in the case of non-spherical or diverse shaped nanoparticles. However, TEM is one of few techniques which can deal with non-spherical particles analysis as there are no assumptions of sphericity inherent in the size calculations. However, due to sample preparation, nanoparticles cannot be observed in suspension, so if TEM is the only method used [83] the behaviour of the particles in suspension remains largely unknown. Still, in some cases TEM can be used to detect nanoparticle dissolution, agglomeration, and reprecipitation [10]. Another issue arising with TEM is a statistical issue- in order to properly assess the size and size distribution several thousand particles need to be analysed, which may be time consuming.
- Sedimentation/centrifugation methods, such as DCS which separates particles in a gradient based on centrifugal force. This method is excellent for complex biofluids such as protein clusters, which typically have quite different density than nanoparticles and thus separation of similarly sized objects of different density is possible. This method gives effective sizes of particles with a biomolecular corona [84]. It could also be used for a dissolution studies. Again, it is suitable mainly for spherical particles, although there is an ellipticity parameter, but limited evaluation of its validity has been undertaken to date.
- UV-Vis based methods (e.g., [27,45]) which utilise the shift in the adsorption maximum as an indicator of particle size. These are effective for metallic particles, such as gold and silver, but are less suitable for non-metallic ones. Also, it is clear that interactions with ions and biomolecules can affect the peak so care in data interpretation is needed. These methods could also be used for dissolution studies.
- Single (Nano) Particle Inductively Coupled Plasma Mass Spectrometry (SP-ICP-MS [43]) which measures the metal ion plumes produced by single particles vaporised in the plasma each second (compared to the measurement in millions of droplets in conventional ICP-MS). Thus, the signal is discontinuous in experimental time, and the mass of the particles determines the peak height and the particle concentration determines the number of peaks per minute. As only one element can be measured at a time, the size of, for example, nanoscale AgCl particles will be interpreted as being much smaller than metallic silver nanoparticles.

Often several methods of size measurements are used in parallel [45]. Contradictory data can be explained by different sample preparation protocols, different base of size distribution (mass, number) and different underlying measurement principles and assumptions [45].

As real samples often have a certain degree of complexity and usually are not monodisperse it might be the most straightforward approach to combine separation techniques such as AF4 (asymmetric flow field flow fractionation) with detectors like DLS or ICP-MS. (AF4) is a state-of-the art method for fractionation and separation of particles in solution. AF4 is often considered for “unusual” or “unique” situations, or for samples that are difficult to separate using other methods. It has also been used to study aggregation of particles in a solution. A limitation of all of the sizing methods described above is that they work best with so called pristine samples and simple fluids. Thus, characterisation of nanomaterials as they exist in products or in the various environmental compartments or *in situ* in living entities is extremely challenging. Indeed, this is one of the key challenges facing the implementation of the European Commission’s regulatory definition of a nanomaterial [85,86].

### 5.2. Surface Area

As surface area is often considered as one of the main factors in nanoparticles toxicity [61], it is surprising that it is assessed only in a very few biological studies [79]. However, at present there is no widely available method to measure this in solution or in complex matrices, although approaches based on NMR are currently being assessed for their general applicability. Thus, standard approaches currently estimate surface area in solution from the dry state. However, it may be possible to estimate surface area from particle primary size and size *in situ* in exposure media. Polydispersity will play a significant role here since a large number of particles smaller than the mean will increase the total surface area (as volume and surface are scale differently) [87].

Another common issue is the high polydispersed silver nanoparticles [45,64,88]. For polydisperse samples it is difficult to separate the effects of different size fractions of particles, which may further blur an already unclear picture. On the other hand, under realistic environmental conditions organisms are exposed to a mixture of different sizes of nanoparticles, as well as to different shapes and compositions [10].

Silver nanoparticles used in biological experiments may represent a mixture of shapes, even if this parameter has not considered as important for their studied properties to date [89].

### 5.3. Coating and Surface Charge

As ‘bare’ silver nanoparticles are unstable in suspension (e.g., [90]), different kinds of coatings are used to decrease agglomeration [81] and dissolution of nanoparticles [78] and to modify their biological activity [23,78,81]. Biological effects of silver nanoparticles are often found to be dependent on their coating [64,78,91]), and as such coatings must be described and considered in the interpretation of biological data.

The potential impact of different contaminants, which can be potentially present in silver nanoparticles, their coatings and suspensions, is rarely studied. According to Samberg *et al.* [92], the way in which a silver nanoparticle suspension is prepared can influence its antimicrobial properties. Unmodified silver nanoparticles, synthesized by base reduction and containing formaldehyde were much more toxic to all studied bacteria strains than “washed” silver nanoparticles (synthesized in the same way, but washed 20 times in phosphate buffer, which decreased the content of formaldehyde). This is an example of where a chemical toxicity is amplified by localization on the nanoparticle surface, and shows the importance of testing dialysate or wash water in parallel with nanoparticle suspensions to identify any additional sources of toxicity not related, or indirectly related, to the presence of the nanoparticles.

Different coatings may also result in different surface charges [91]. As surface charge may influence nanoparticle interactions [59] with living systems and thus their toxicity, and because it may change depending on the dispersant [81] it should be measured in the medium used in the experiment. In some studies, no impact of zeta potential on toxicity was found [78]. On the other hand, Suresh *et al.* [91] suggest a direct correlation between the cytotoxicity of dispersed silver nanoparticles and their overall surface charge and/or surface coating.

In some cases, even where the surface charge of silver nanoparticles is used to explain some of their interactions with cell, it is not actually measured [59], which leaves the data open to interpretation, and weakens the strength of the conclusions.

## 6. Transformation of Silver Nanoparticles in the Environment

The behaviour of silver nanoparticles in solution is difficult to analyse due to the complex chemistry of silver in aqueous solutions [55,81,93] and others) and to the variety of published results (different media, different shapes, sizes and coatings on the nanoparticles). A selection of relevant studies from recent years is presented in Appendix 1. The stability of silver nanoparticles strongly influences their toxicity, as silver ions are considered to be one of the main silver toxicity factors [78]. Also agglomeration of silver nanoparticles was reported to influence their toxicity [91].

Factors such as ionic strength and composition [70,78,81,82,94], pH [11,82,93], dissolved organic matter [58,81,93], humidity of the environment [10], dissolved oxygen concentration [91,93], temperature [69,93], size, shape and coating [69,70,78,81,82,91,94] of nanoparticles and their concentration [82] all influence their stability. To increase nanoparticles stability and to avoid uncontrolled agglomeration in medium, the dispersion should be prepared very carefully [79], and prepared freshly and in an identical manner for each experiment, and the dispersion characterized in parallel with the toxicity assay.

Given the increasing amount of surface area as particle size decreases, nanoparticles have high surface energies. In specific environmental conditions silver can be oxidized and dissolved to silver ions, which are the main active and reactive species of silver. Due to the limited specific surface area of bulk silver the amount of ionic species available is usually quite limited. As the particle size decreases towards nanoscale (according to CEN ISO/TS 27687:2009 a nanoparticle is an intentionally manufactured particle, which in at least one dimension is in the range of 1–100 nm), this situation is reversed, with a much higher proportion of the atoms being at the surface and consequently greater propensity for dissolution, or reaction. Also, the increased surface curvature and gaps in crystall lattice result in increased reactivity. Thus, the physico-chemical properties of nanomaterials are different to those of the corresponding bulk materials. 

In some recent studies no clear, nano-specific effects were found. Ma *et al.* [95] did not find any lattice strain even in silver nanoparticles as small as 6 nm. According to the authors increased solubility of smaller nanoparticles can be explained solely by the modified Kelvin equation. Xiu *et al.* [96] ruled out direct particle-specific antibacterial activity of nanosilver, and stated that silver ions are the definitive molecular toxicant.

Although the release of silver ion is considered as one of the main factors in silver nanoparticle toxicity [94], studies to determine the dissolution rate of silver nanoparticles in media and the impact of silver dissolution rate on cells/organisms are quite rare [78,82]. With decreasing size of silver particles, the potential for releasing silver ions increases and moves from the silver sulphide extreme (minimal release) toward silver nitrate extreme (maximal release) [1], although the particle coating also plays in role in the process [78]. The behaviour of silver nanoparticles (polymer-coated and with unspecified coating) in two simulated biological fluids representative of the fluids present in lungs, as inhalation is regarded as important uptake route in humans [42], was studied by Stebounova *et al.* [82]. The results showed that the initial concentration of nanoparticles has a significant impact on their stability and sedimentation. The authors use Dejaguin–Landau–Verwey–Overbeek (DLVO) theory as a basis for theoretical calculations explaining nanoparticles behaviour in solution, and their experimental results concur with the theory used. According to DLVO theory, the stability of particles is determined by the net electrostatic surface interactions of the particles and their Van der Waals forces. Polymer-coated silver nanoparticles (with higher surface charge) were more stable than the other studied type (with unspecified coating) in both water and simulated biological fluids. In artificial lysosomal fluid (ALF) both nanoparticles were less stable than in water, probably due to the higher ionic strength of the solution. None of the studied fluids contained organic matter (*i.e.*, proteins, lipids *etc.*), which may strongly influence nanoparticles behaviour in suspension [58,81,93] so the results of this study may not be relevant for “proper” biological fluids.

In the study of Yang *et al.* [78] the impact of dissolved silver and particle coating on *Caenorhabditis elegans* was investigated. The authors used three complementary approaches: pharmacological (rescue with trolox—ROS scavenging chemical and *N*-acetylcysteine-chelating chemical and an antioxidant), genetic (analysis of metal-sensitive and oxidative stress-sensitive mutants) and physicochemical (including analysis of dissolution of silver nanoparticles). The analysis of nanoparticle behaviour in suspension was investigated in detail. It was found that lower ionic strength medium resulted in greater toxicity (measured as growth inhibition) of all tested silver nanoparticles: the EC50 values (see Box 2) and threshold lethal doses for each nanomaterial were 1.5 to 12 times lower, for AgNO_3_ 100 times lower than the same nanoparticles in a higher ionic strength medium. Such an effect might be caused by the 1600 times higher concentrations of chloride in the medium of higher ionic strength and the 10 and 3.5 times higher levels of Ca^2+^ and Mg^2+^, respectively. The presence of divalent cations can greatly influence nanoparticles agglomeration and dissolution (and thus decrease their bioavailability). Chloride ions can bind silver ions and form poorly soluble AgCl complexes that precipitate from solution, thus reducing the concentration of available ionic silver.

Yang *et al.* [78] found a linear correlation between silver nanoparticle toxicity and the amount of dissolved silver. Oxidative dissolution was limited (maximally 15% in 24 h), but still crucial for the toxicity of all studied silver nanoparticles. In the case of the less soluble silver nanoparticles, oxidative stress, an effect specific to nanoparticulate silver, could be observed. The toxicity of silver nanoparticles was never greater than would be predicted by complete dissolution of the same mass of silver as silver ions.

In the kinetic experiments in deionized water conducted by Lee *et al.* [70], 36% of sol-type, citrate-stabilized silver nanoparticles and powder-type silver nanoparticles were converted to silver ions. Equilibrium was reached within the first 6 h of the 7 day experiment (in the same, unchanged solution). The dissolution rate of non-stabilized powder-type silver nanoparticles was expected to be higher, and in more complex medium used for ecotoxicity tests the dissolution rates for sol-type and powder-type silver nanoparticles were significantly different (11% and 49% respectively). Silver ion concentration in silver nanoparticle suspensions reached an equilibrium concentration after 48 h, which was the exposure time of the acute aquatic toxicity test (conducted on an aquatic organism, *Daphnia magna*). The 48-h EC50 values for *D. magna* of powder-type silver nanoparticle suspensions were 0.75 μg/L total Ag. For sol-type silver nanoparticle suspensions, the 48-h EC50 values for *D. magna* were 7.98 μg/L total Ag. The EC50 values for the dissolved silver of powder-type and sol-type silver nanoparticles showed similar results (0.37 mg/L and 0.88 mg/L, respectively) despite their different EC50 values in total Ag. For freshwater fish and invertebrates, a key mechanism of acute silver toxicity consists of reduction of Na^+^ uptake by the blockage (and thus impaired function) of gill Na^+^/ K^+^-adenosine triphosphatase channels [70].

In the study of Liu *et al.* [93] the aim was to investigate the kinetics of silver ion release from nanoparticles. As stated by the authors, silver ions are released during a cooperative oxidation process, during which both dissolved oxygen and protons are required. Peroxide intermediates are released during this process, which, under some conditions, may lead to complete dissolution. Ion release increases with temperature in range 0–37 °C and decreases with increasing pH or addition of humic or fulvic acids. Even at the lowest pH studied (pH = 4.0) the dissolution process requires dissolved oxygen. Argon-stripping of dissolved oxygen, addition of organic matter (humic or fulvic acids), addition of stabilizing citrate, temperature reduction or increase of pH can decrease the dissolution rate of silver nanoparticles. The authors performed their study using only one type of citrate-stabilized, small (4.8 ± 1.6 nm) silver nanoparticles.

In another study [94] a detailed, systematic assessment of silver nanoparticle dissolution in biological medium, including thermodynamic calculations of silver species partitioning, the rates of oxidative silver dissolution for nanoparticles and macroscopic foils and unified area-based release kinetics was performed. Several chemical approaches were used to control silver ion release. Thiol and citrate ligand binding, formation of sulfidic coatings, and the scavenging of peroxy-intermediates significantly slowed the silver ion release. The release was accelerated by peroxidation or particle size reduction. The silver ion release profile could be modified by polymer coatings with complexation sites. The approaches used allowed the authors to tune the antibacterial properties of silver nanoobjects.

The influence of various solution compositions on dispersion stability of concentrated silver nanoparticles (30–50 nm in size) behaviour in various electrolyte concentrations (1 or 100 mM NaNO_3_) was studied by Chappell *et al.* [81]. Generally the quality of the dispersion was greatly impacted by the electrolyte concentration. Only the non-ionic surfactant (BRIJ 35) provided excellent dispersion of silver nanoparticles (unaffected by electrolyte concentration), while EDTA and a large polysaccharide (alginic acid) did not work well as chemical dispersants (surfactants). In most of the studied systems, the addition of organic stabilizers caused supersaturation of dissolved silver, and the authors concluded that, regardless of the stabilizing effects of humics, polymer loading may enhance the dissolution and release of dissolved silver into the environment. Sorption of organic compounds onto the surface of silver nanoparticles was expected to affect the dynamics of nanoparticle dissolution, depending on the thickness and conformation of the sorbed layer. If the nanoparticles were completely covered, van der Waals attractive forces would be shielded, which would prevent particle agglomeration. Sorbed compounds would modify the surface characteristics of nanoparticles (especially in the case of charged compounds) and alter the possibility of nanoparticle-surface interactions with the medium components. Sorption of hydrophilic groups may thus slow down agglomeration of nanoparticles, while more hydrophobic compounds may promote destabilization of the dispersion.

The stabilizing effect of organic compounds was also studied by Khan *et al.* [58]. Silver nanoparticles (around 65 nm in size) in water were unstable (zeta potential close to 0). In the presence of bacterial exopolysaccharides (EPS) negative surface charge increased to higher than −30 mV and the agglomeration was reduced. EPS adsorption was dependent on pH, salt concentration and EPS concentration. In a similar study, the release of ~15% of total mass of silver was found after the dispersion of silver nanoparticles in growth medium containing EPS (EPS were found on the surface of nanoparticles) [88]. In the study of Yang *et al.* [78] free citrate present in citrate-coated silver nanoparticle suspension complexed a large portion (42%) of the dissolved silver.

In the study of Unrine *et al.* [97] aggregation and dissolution of two types of silver nanoparticles (PVP and gum arabic coated) was investigated in four different aquatic microcosms: surface water; water and sediment; water and aquatic plants; or water, sediment, and aquatic plants. It was found that plants responded to Ag exposure by releasing dissolved organic matter, which was in turn binding silver ions. Dissolved organic matter had a stabilizing effect on PVP-coated nanoparticles, but it caused dissolution and removal of gum-arabic coated nanoparticles, which were found to overcome significant chemical transformation (22%–28% of the particulate Ag associated with thiols, 5%–14% present as oxides).

In the studies cited above, interactions with organic matter caused both increased dissolution and stabilization of silver nanoparticles. In the study of Quik *et al.* [98] heteroaggregation of CeO_2_ nanoparticles with the solid fraction of natural colloids found in unfiltered river water was the main mechanism causing sedimentation of the nanoparticles, as long as the nanoparticle concentration was low (1 mg/L). In more concentrated suspensions, homoaggregation played more important role.

**Figure 2:**
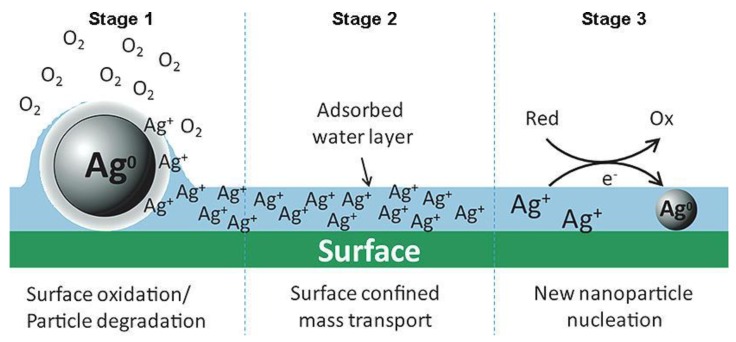
Proposed pathway for new particle formation from parent (nano) particles. Reprint with permission from [10]. Copyright 2011 American Chemical Society.

As observed by Glover *et al.* [10] relative humidity has a great impact on silver nanoparticles transformation. Under ambient conditions at relative humidities greater than 50%, new nanoparticles were formed near the parent ones. Such a phenomenon, which was strongly humidity-dependent, was observed for many different kinds of silver nanoparticles, with many different coatings. The authors proposed a three-stage model, which explains the formation of new nanoparticles (Figure 2). Both chemical and photo reduction seemed to be crucial for the last stages of the process. For example, particles stored for three weeks at 0% relative humidity remained unchanged, but the same particles stored under humid conditions showed dramatic morphological transformation. Changes in shape, size and number of nanoparticles could be observed within a few hours. It was found that access of light also had an influence on the extent of silver nanoparticle degradation and re-formation (photoreduction processes). Due to the last observation, nanoparticles transformation was also studied without using a TEM electron beam (by atomic force microscopy), but the results appeared to be very similar. The potential catalytic impact of positively charged hydrophilic surfaces of TEM grids was ruled out by additional experiments, in which neutral, mica surfaces were used.

The formation of new silver nanoparticles occurred also when common silver objects (jewelry, silver wire, silver spoons and forks) were exposed to humid conditions. The described phenomenon can be more general, as experiments with copper objects gave similar results. It can be concluded then, that under certain environmental conditions (high humidity, presence of light) many metal objects may be potential sources of nanoparticles.

In the study of Akaighe *et al.* [99] formation of silver nanoparticles via reduction of silver ions was observed in the presence of humic acids under environmentally relevant conditions. Humic acids obtained from Suwanee River and sedimentary humic acids were found to reduce silver ions at 22 °C, whereas humic acids from soil samples reduced silver ions only at much higher temperatures (90 °C). Silver nanoparticles were detectable after 2–3 days (at 22 °C) or 90 min (at 90 °C). Newly formed nanoparticles were usually spherical and had a very polydisperse size distribution.

According to Adams and Kramer [55] the formation constant for reaction of Ag^+^ and HS^-^ is high (logK ≈ 13) (formation constants of similar magnitude have been determined for several organic sulphide compounds, like thiols), so silver (I) complexes with sulphides are very strong. The formation constants for compounds containing oxygen and nitrogen groups are much lower (e.g., logK ≈ 6 for EDTA). Thus, as long as sulphide is in excess of silver, silver sulphide complexes will dominate silver speciation (so the free ion concentration is negligible). Inorganic sulphide was found even in fully oxic surface water (in submicromolar concentrations), probably in the form of colloidal metal sulphides (stable under oxidizing conditions for periods of several hours). In surface water silver is present in very low concentrations (<nM), so inorganic sulphide will be always in excess (200 to 300 times in excess in wastewater effluent and receiving waters, 1000 to 15,000 times in excess in pore waters). Thiols were present only in pore waters (in low nanomolar concentrations) and do not seem to play any role in silver speciation in fresh waters. The authors do not include Cl^−^ in their calculations. The formation of silver-sulphur complexes in mopping up ionic silver is considered as important also by other authors. According to Kaegi *et al.* [45] metallic silver nanoparticles were transformed into Ag_2_S within 2 h (in a non-aerated tank). The formation of silver salts of very low solubility (AgCl, Ag_2_S) significantly decreases the bioavailability of silver ions and reduces the toxicity of silver in solution [45], but, as stated by Adams and Kramer [55], despite the strength of silver-sulphur (II) complex formation, silver is labile between sulphur (2II) species (e.g., sulphides and thiols), so there can be an exchange between an inorganic sulphide species in the aqueous phase and thiolic sites in the organism during uptake processes or following localization in living entities. According to Kaegi *et al.* [45] effective silver nanoparticle transformation into Ag_2_S was caused by high sulphide levels in non-aerated mixed liquor in a pilot WWTP. Complete silver nanoparticle transformation did not occur (even in the presence of sufficient sulphide). The authors conclude that a core-shell structure with a passivating Ag_2_S surface layer was formed at the nanoparticle surface.

In the study of Glover *et al.* [10] newly formed silver nanoparticles containing metallic silver were observed under high humidity conditions, and no evidence of Ag_2_S or AgCl was found (although nanoparticles were kept in water or, during the experiment, on TEM grids, so there was no source of Cl^−^, S^2−^ and other ions). In the experiments conducted by Choi and Hu [59] the chloride and sulphide concentrations in medium were kept low to avoid silver salts precipitation (in experiments where the effect of silver ions was investigated). Despite this, it is hard to state that the concentration of added silver ions remained unchanged (as it may form insoluble complexes even with low concentrations of chloride and other ions).

According to Yang *et al.* [78] 89% to 96% of the silver present *in vivo* (so, presumably, originating from administrated silver nanoparticles and ions), is complexed with sulphur atoms *in vivo*. However, there is also emerging evidence of formation of nanoparticles *in vivo* potentially as a protective mechanism, e.g., a study of the magnetic properties of superior temporal gyrus brain tissue from 11 Alzheimer’s disease and 11 age-matched control subjects demonstrated an exponential correlation between the concentrations of Fe{2+}-ion-containing iron oxide, magnetite and the fraction of those particles that are smaller than 20 nm in diameter. These data provide circumstantial evidence in favour of their genesis within the 8 nm diameter cores of the iron storage protein ferritin [100]. Given the evidence of formation of small silver nanoparticles under high humidity conditions, such a mechanism could potentially occur also for silver *in vivo*. Liu *et al.* [93] investigated another mechanism of silver ion concentration depletion, which may play a role in more concentrated silver nanoparticle suspensions. The authors paid attention to the absorption of silver ions onto the surface of nanoparticles, which resulted in a complex mixture of Ag^0^, free and complexed silver ions and surface absorbed silver ions in the nanoparticle suspension. In the case of small (2–8 nm) silver particles with a very high surface area, the amount of ions that bind to the particles surface is so high that it causes a detectable decrease of free silver ion concentration and a shift of the nanoparticles surface charge to more positive values. In another study [94] a thermodynamic calculation of silver species partitioning in biological medium containing different NaCl concentrations was performed, and the silver ion concentration were found to drop rapidly with increasing chloride concentration. However, further increase of chloride content (beyond 2–3 mM, which matches the Cl^−^ concentration in mitochondria, cytoplasm or extracellular spaces) caused an increase of AgCl*_x_*^1−*x*^, which enhanced the amount of total dissolved silver. Silver salt addition to biological media causes rapid precipitation of AgCl nanoparticles, which is followed by dynamic aggregation, settling, and cellular uptake, or even photoreduction—none of those potentially competing phenomena are fully understood making data interpretation difficult. On the other hand, the addition of metallic silver nanoparticles causes gradual ion release.

One of the central questions in understanding silver nanoparticles behaviour in suspension is do they persist under realistic environmental conditions? According to thermodynamic and kinetic models [93] silver nanoparticles are not persistent in environments containing oxygen, which appears to answer the question as to whether special regulations concerning silver nanoparticles are needed, or whether regulation can be based on existing data on conventional forms of silver. However, systemic studies are needed to understand the role of surface coatings and interactions with (environmental) biomolecules on the kinetics of silver dissolution and the new species formed under different environmental conditions. There is sufficient evidence that adsorbed species can significantly prolong the particle nature of silver nanoparticles under environmentally relevant conditions [45] for these issues to be more fully considered.

A significant rate of dissolvation and release of silver ions under different conditions was proved by many studies [10,45,78,88] although some authors stated that the dissolution rate is very low [45,79,82]. According to Cao *et al.* [31], only a minimal leaking of silver ions from Ag-PIII-originated surfaces (silver nanoparticles 5–8 nm in size, made using a single step silver plasma immersion ion implantation method) samples was found, even after 60 days at 37 °C (unknown oxygen concentration in the solution).

Kittler *et al.* [69] also investigated the processes of silver nanoparticle dissolution. Citrate and PVP-coated 85 nm silver nanoparticles were dispersed in water and stored up to 125 days. Although the conditions may not be relevant for *in vitro* biological experiments, we should keep in mind that silver nanoparticles are often stored dispersed in water; water is also the main medium nanoparticles have contact with in the natural environment. Dissolution experiments were performed via dialysis in a 100-fold excess volume, and such conditions could increase the dissolution rate. Diffusion of silver ions through the dialysis membrane was much faster than the dissolution process and can be neglected as a contributing factor to the overall rate. In all cases a limiting value of the released silver ions was observed. Even if some nanoparticles released up to 90% of their weight into water, the dissolution was never complete. The final degree of dissolution did not depend on the absolute concentration of nanoparticles (final concentration of silver ions was not constant), but seemed to be characteristic for certain nanoparticles. The rate of dissolution was found to be temperature-dependent. The dissolution rate was higher for PVP-stabilized nanoparticles than for citrate-stabilized nanoparticles [69]. The authors speculate that this could be due to the citrate reductive capability (as during the synthesis). The results of biological studies on human mesenchymal stem cells showed that silver nanoparticles which had been stored in dispersion for several weeks were considerably more toxic than freshly prepared dispersions (due to the increased concentration of silver ions). Those results underline the importance of the “age” of silver nanoparticles dispersions used in biological experiments. Aged silver nanoparticles (e.g., after 1 or 6 months of immersion) caused complete cell death. On the other hand, 3-day “old” dispersions reduced cell viability to 70%. Six-month “old” dispersions had a lethal concentration that was ~20 times lower than that of the freshly prepared dispersion. Based on the above-cited results, time-dependent dissolution leading to higher toxicity seems to be evident, although again this might be dependent on the synthesis route, and the capping or stabilizing layer. However, it is clear that literature reports should also make some comment (where possible) on the “age” of their samples at the time of the study.

Kittler *et al.* [69] also analysed the possible fate of silver ions in solution. The released silver ions can be bound by proteins (in biological media) or precipitate as insoluble silver salts (e.g., AgCl or Ag_3_PO_4_). Both processes reduce the silver ion toxicity, so consequently the toxicity of “aged” silver nanoparticles is not as high as could be expected from the release curves. This also suggests a possible mechanism for mitigating the effects of silver nanoparticle dissolution during storage, as dispersion media could be modified to also contain salts or proteins to “mop up” the released silver ions, or samples could be treated with such salts immediately prior to toxicity assessment experiments. Another approach could be to purge the stocks with Ar (or N_2_) and then store the stock suspensions under anoxic conditions. As the dissolution of Ag^0^ is coupled to an oxidation reaction, the removal of the oxidation agent (in that case O_2_) should stop the dissolution.

As can be concluded from the studies discussed in this section, transformation of silver nanoparticles by their environment significantly influences their chemical composition, bioavailability and (eco) toxicity. It is of particular concern how those transformations influence the impact which silver nanoparticles have on humans. It can be assumed that the impact of silver nanoparticles on human cells and tissues could be significantly different depending on the route of uptake as this will determine the biomolecules that coat the nanoparticles (see next section for details). When particles get into the human body directly (via catheters, wound dressings, *etc.*) they interact with human cells and tissues in their original, unchanged form. The situation is radically different for silver nanoparticles released from textiles and other consumer products, which have undergone environmental transformation prior to uptake by humans or other organisms. The second scenario has not been generally studied *in vivo* or *in vitro* (except via some ecotoxicological studies).

In the study of Shoults-Wilson *et al.* [46] the effect of surface coating on silver nanoparticles toxicity in soil was investigated. Earthworms (*Eisenia fetida*) were used as a model organism. Silver nanoparticles were found to be much less toxic than silver nitrate (used as a control) but no significant impact of nanoparticles’ coating (PVP and oleate) was found. Earthworms were found to accumulate significantly more Ag when exposed to silver nitrate than to silver nanoparticles, although no significant differences between silver tissue concentrations after the exposure to the two studied types of nanoparticles were assessed. This is an area of active research, and significant additional reports on this aspect are expected soon.

In the light of the studies cited in this section (along with many others) it can be questioned if nanosilver, after being released into the environment, can still be considered as an engineered nanomaterial as the particles lose their original functionality and properties. It is clear that more working comparing “aged” and fresh samples are needed, as well as a convention in literature to report sample “age” or dissolution state at the time of performing experiments, although how that would be achieved has yet to be determined and would require some method development. Additionally, it is still a subject of a discussion if, for example, rich sets of data from the photographic industry (in which silver compounds have been used for a long time) can be used in assessing the risk imposed by nanosilver.

### 6.1. Transformation of Silver Nanoparticles in Living Systems

It is widely accepted that nanoparticles, as well as other materials, are covered with proteins and other biomolecules immediately after contact with a biological medium [101]. This “protein or biomolecule corona” formation was studied for a range of nanoparticles (e.g., polystyrene, silica, gold *etc.*), but its impact on silver nanoparticle (and other forms of silver) interactions with cells and organisms still needs investigation.

Powers *et al.* [64] found that removing serum from the cell culture medium enhances the impact of silver ions. It was hypothesized that serum proteins bind silver ions and thus decrease their availability and thus toxicity. No corresponding effect was found for citrate-coated silver nanoparticles.

Peptide-coated silver nanoparticles were used in the study of Haase *et al.* [102]. The peptide, covalently bound to the nanoparticles’ surface, enabling a tight size and shape control of the synthetized nanoparticles. The peptide-stabilized nanoparticles were found to acquire a complex protein corona, enriched with some serum proteins, the composition of which was quite different to the protein corona formed on the same nanoparticles synthesized without peptide.

Based on the results of experiments on negatively charged gold nanoparticles (2–4, 5–7 and 20–40 nm) and positively charged silver nanoparticles (of similar size) and differences in their uptake by macrophages, Yen *et al.* [76] speculate that negatively charged gold nanoparticles might adsorb serum protein and enter cells via the complicated endocytotic pathway (as well as via the pinocytotic pathway), which results in higher cytotoxicity and immunological response of gold nanoparticles as compared to silver nanoparticles. Surface charge was measured in undetermined medium, and it is worth noticing that according to other studies [103] nanoparticles’ surface charge changes after the dispersion in medium containing proteins. The authors, based on TEM images, suggest that silver nanoparticles did not absorb serum proteins due to their small surface charge, and that those nanoparticles entered the cells only by the pinocytosis pathway.

In the study of Sun *et al.* [27] human serum albumin (HSA) was used to stabilize newly synthetized silver nanoparticles. Incubation with HSA did not decrease the antiviral properties of the studied nanoparticles.

Other authors [104] identified a number of proteins from *Escherichia coli* that bind specifically to silver nanoparticles (average size ~30 nm). Tryptophanase (TNase) was observed to have an especially high affinity for both studied surface modifications (carbonate-coated and bare surface) despite its low abundance in the bacteria. As found in the study, purified TNase loses enzymatic activity upon associating with silver nanoparticles, which indicated that the active site may be in the vicinity of the site(s) of binding to the nanoparticle surface. Preferential binding of some protein fragments based on surface coating was also found. Silver adducts were identified for some fragments and were found to be characteristic of strong binding to silver nanoparticles, but not of association of the fragments with silver ions. The results highlight the potential effect of nanoparticle surface coating on bioavailability.

In the recently published study of Liu *et al.* [11], a silver nanoparticle transformation pathway within a living organism is proposed. The authors state that nanosilver can be at least partly dissolved by gastric fluid (of very low pH). The resulting soluble silver species (mainly ions) form complexes with proteins (via thiol groups) and can be circulated systemically. As calculated from the ion concentrations in body fluids, insoluble silver salts, like AgCl, should not precipitate in the bloodstream. Such insoluble silver species may be found in toxicity studies, where the doses of silver are much greater than those expected under realistic natural conditions. The authors pay great attention to the fate of silver complexes within the body. First, they observed that silver-thiol complexes can be photoreduced to metallic silver (e.g., in skin, which leads to agryria where the skin becomes blue or bluish-grey coloured). While silver can easily exchange between thiol groups (even though the solubility of those complexes is very low), reduced, metallic silver is immobile. Those newly formed, “secondary” silver nanoparticles can react with sulphides present in body fluids via reactions resembling those known from the transformation processes occurring in the environment. The authors found also that in body fluids sulphur can be replaced by selenium (this replacement is thermodynamically favourable), which explains the presence of selenium in silver deposits in skin, which are found in patients with agryria. This study reveals several new processes of silver transformation within living system.

Also in the study mentioned above, slow dissolution of silver nanoparticles in other artificial biofluids is explained not only by higher pH, but also by the presence of BSA, which may protect the nanoparticles from dissolution processes [11].

Formation of a protein corona and the consequences of this for bioavailability and uptake of nanoparticles is not the only nanoparticle-protein interaction which is relevant from a biological point of view. Nanoparticles were shown to affect the conformation of some proteins bound to their surface, which may lead to denaturation of some bound proteins or may increase their stability/activity [101]. The influence of nanoparticles on protein fibrillation has also been shown [105]. According to Shemetov *et al.* [106], interactions of proteins with nanoscale objects may lead to abnormal conformational changes and, for example, exposure of cryptic epitopes or self-assembling on nanoparticle’s surface. Those phenomena were studied on a range of different nanoparticles, also some metallic ones [107,108]. As can be concluded from the known results, a nanoparticles’ impact on proteins depends mainly on their size (and thus surface curvature) and surface properties (surface charge). Thus, the impact of silver nanoparticles on proteins can be significant: as shown by Shrivastavaa *et al.* [109], silver nanoparticles can significantly retard polymerization of fibrinogen and prevent clot formation (possibly due to inhibition of the enzymatic activity of thrombin). Still, the lack of relevant studies make it difficult to understand if some properties of silver (e.g., the release of silver ions) play a significant role in (or are influenced by) protein corona formation and other protein-nanoparticle interactions. Several studies suggest that dissolved organic matter (in many cases containing proteins and/or polysaccharides) significantly influences silver nanoparticles stability [58,81,93], but there is still not enough information regarding the impact of silver nanoparticles on protein stability.

## 7. Mechanisms of Silver Nanoparticle Action in Bacteria and Potential for Bacterial Resistance

The antimicrobial activity of silver in general, and of silver nanoparticles in particular, is of significant interest because it appears to be independent of the strain of bacteria. Crucially, antibiotic resistant strains, MRSA (methicillin, or multiple-resistant *Staphylococcus aureus*), *E. coli* O157, and others are affected by silver [6]. The reasons for this are not fully clear as yet, but could be related to mechanisms of silver ion action on bacteria [110], trypanosomes and yeasts, all of which can take up and concentrate silver (and copper) from dilute solutions in sufficient amounts to lead to lead to saturation of all enzyme-protein molecules per cell [6]. Other effects observed include structural changes in bacterial cell walls and intracellular and nuclear membranes as well as bacterial DNA and RNA denaturation, inhibiting replication [6,110,111]. Possibly these effects in bacterial RNA and DNA are related to (or in addition to) the observed effects on mitochondrial respiration and cytosolic protein that lead to bacterial cell death. The distinct activity of silver ions, rather than nanoparticle derived impacts, has not been understood as yet. Ovington [111] noted that nanocrystalline silver products (Acticoat^®^, Smith and Nephew) can release a cluster of highly reactive silver cations and radicals, which provide a high antibacterial potency.

Li *et al.* [29] point out three possible antibacterial mechanisms of silver nanoparticles:

(1) Adhesion of nanoparticles to the bacteria surface, altering the membrane properties. The small size and extremely large surface area of nanoparticles enables them to make strong contact with the microorganism surface [5]. As stated by Cao *et al.* [31] who studied the antibacterial properties of silver nanoparticles embedded in titanium (Ag-PIII-originated surface), the attachment of bacteria to such a surface correlates with the surface zeta potential of the nanoparticles. All studied Ag-PIII surfaces reduced the proliferation of both types of bacteria studied (Gram-positive *Staphylococcus aureus* and Gram-negative *Escherichia coli*).
(2) Silver nanoparticles penetrating inside the bacterial cell, resulting in DNA damage. In the study of Choi and Hu [59] the inhibition of nitrifying organisms was correlated with the fraction of silver nanoparticles less than 5 nm, which was more toxic than any other form of silver (silver ions, AgCl colloids). The authors suggest that this may be due to easier (active) transport through the cell membrane of uncharged silver nanoparticles than of charged silver ions.
(3) Dissolution of silver nanoparticles releases antimicrobial Ag^+^ ions which can interact with sulphur-containing proteins in the bacterial cell wall, which may lead to compromised functionality. This phenomenon is often considered as the main mechanism of the antimicrobial activity of nanosilver [6,111,112], so we can presume that the vast knowledge of antimicrobial properties of silver ions can be applied to the nanosilver case. At the same time, the problem of bacterial resistance to silver ions remains meaningful for at least some usages of silver nanoparticles. Interaction of dissolved Ag^+^ ions with cell wall and cytoplasmic proteins was also proposed by Cao *et al.* [22], who also highlight the fact that silver ions interaction with the thiol group of vital enzymes may result in their impaired function or inactivation. The exchange of silver ions between inorganic sulphur complexes and thiols was also proposed by [55,89] and others. Disruption of respiration and establishment of proton motive force as an effect of interactions with thiol groups of enzymes and other proteins is also stated by Hall Sedlak *et al.* [113]. According to Lee *et al.* [70], silver ions inhibit enzymes acting in the phosphorus, sulphur, and nitrogen cycles of nitrifying bacteria*.* Silver ions can enter from the environment or originate from sustained dissolution of silver nanoparticles taken up by bacteria.

According to Samberg *et al.* [92], the antibacterial activity of silver ions is caused by the synergistic effect between the binding of silver ions to the cell wall, their uptake and subsequent accumulation in the cell, and their interference with critical biomolecules within the cell. According to the authors, the steady release of silver ions from the degradation of silver nanoparticles is a critical function of silver nanoparticles that should be considered prior to synthesis (as it depends on the coating and synthesis method).

According to Wong and Liu [5] silver ions can also interact with phosphorus-containing compounds (e.g., DNA). Interference with DNA replication processes, which stops bacterial proliferation and decreases the number of cells over time, is mentioned also by other authors as a consequence of exposure to silver ions dissolving from silver nanoparticles [22].

In contrast, some authors state that silver ions do not play an important role in the antimicrobial mechanism of metallic Ag NPs-modified films coated on titanium or titanium dioxide substrates [22,31]. The creation of free radicals and induction of oxidative stress should be also taken into account following uptake of silver nanoparticles/ions, and is mentioned by many authors [5,22]. Reactive Oxygen Species (ROS) can be generated outside the cell, in medium, or inside the cell, also as a consequence of cell damage/disruption [94,114]. According to Choi and Hu [59] bacterial growth inhibition caused by all studied forms of silver was correlated with intracellular ROS levels. Photocatalytic ROS fraction did not show such a correlation. As the ROS concentrations were different for each of the studied forms of silver, the authors concluded that other sources of toxicity should also be taken into account. Although there was some relationship between the bacterial growth inhibition and silver ion concentration, silver nanoparticles were found to be more toxic than the equivalent concentration of silver ions, possibly due to an increased localized concentration of the ions surrounding the nanoparticles and differential localization as a result of silver nanoparticles being actively transported into cells via receptor mediated processes. The amount of ROS generated by AgCl colloid was comparable to that generated by silver nanoparticles, and the equivalent concentrations of silver ions generated less ROS.

In the recent study of Xiu *et al.* [96], the authors ruled out any particle-specific antibacterial action of nanosilver. By synthesizing and testing different kinds of silver nanoparticles under anaerobic conditions (in which the dissolution processes are stopped), they proved that nanosilver shows no toxicity to *E. coli* when the release of silver ions is ceased. Furthermore, the toxicity of the tested silver nanoparticles followed the dose-response pattern of *E. coli* exposed to silver ions. As revealed by the study, *E. coli* survival rate was higher when bacteria were treated with low, sublethal doses of silver nitrate and silver nanoparticles.

(4) As described in detail by Cao *et al.* [31], the proton electrochemical gradient in bacteria is established and maintained by respiratory processes (net transfer of protons from inside to outside of bacteria). ATP synthesis takes place when protons enter the cell (via ATPase), so the electrochemical gradient is an essential driving force for ATP synthesis in bacteria (similar processes occur in mitochondria). If those processes are interrupted, essential energy for all energy-dependent reactions cannot be provided, which leads to the (microbial) cell death. According to the authors, the proton-depleted regions formed around silver nanoparticles embedded in titanium (due to micro-galvanic effect, which causes proton consumption) may disrupt the electrochemical gradient in the bacteria’s intermembrane space and interfere with adhesion and proliferation [31]. The disruption of transmembrane electrochemical gradient, the importance of which is described above, leads eventually to cell death. As stated by the authors, the hypothesis mentioned above is supported by another study, where proteomic analysis results indicated that silver nanoparticles of average diameter 9.3 nm may accumulate in the protein precursors leading to depleted intracellular ATP levels [115].

The shape of silver nanoparticles can be an important factor in their antibacterial properties. In the aforementioned study of Pal *et al.* [89] truncated triangular silver nanoplates with a {111}-lattice plane as the basal plane displayed the strongest biocidal action against *Escherichia coli* (compared with spherical and rod-shaped nanoparticles and with AgNO_3_). These truncated triangular silver nanoparticles resulted in almost complete inhibition of bacterial growth at a total silver content of 1 μg. The authors speculate that the action of the silver nanoparticles is broadly similar to that of silver ions, and that a bacterial cell in contact with silver nanoparticles takes in silver ions, which inhibit respiratory enzyme(s), facilitating the generation of reactive oxygen species and consequently damaging the cell [89]. A type of surface lattice seems to influence the antibacterial properties of silver nanoparticles, although further studies are needed to confirm the lattice structure facilitates faster dissolution to other forms, or whether other factors contributed the enhanced antimicrobial activity of the triangular particles.

**Figure 3:**
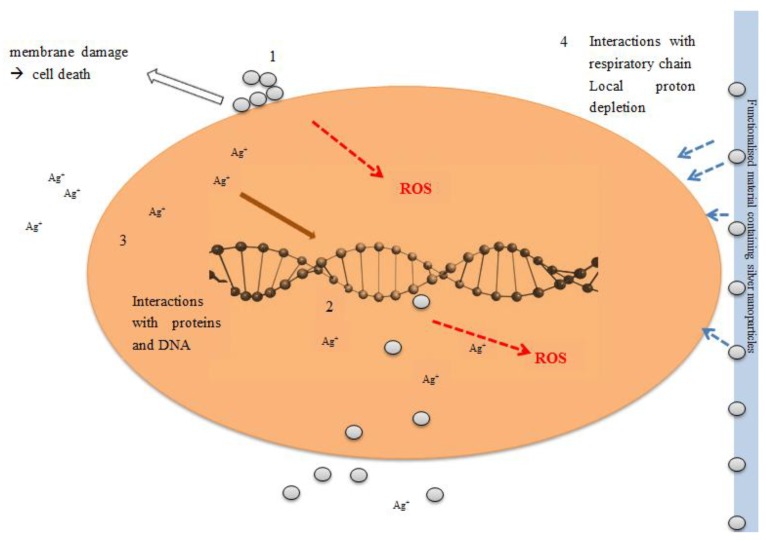
Schematic representation of the known mechanism(s) of antibacterial action of silver nanoparticles and released ionic silver. The numbers 1–4 correspond to the mechanisms described in the paragraphs above. Grey circles indicate silver NPs and Ag^+^ implies ionic silver released from the NPs.

The mechanisms of antibacterial actions of silver are summarized in Figure 3.

Some bacteria have adapted to growth in the presence of high concentrations of silver ions. Those isolates which developed such a resistance in the natural environment utilize efflux pumps [113]. Such a pump may be encoded by plasmid-borne gene cassettes, which could be transferred to other bacterial strains. In the study of Khan *et al.* [88] a silver-tolerant strain of *Bacillus pumilus* isolated from sewage was treated with a high concentration (10–200 mg/L) of silver nanoparticles (10–40 nm according to TEM analysis). Growth kinetics remained similar to the control, but a reduction of the amount of extracellular polymeric substances (EPS) was observed. ESP capping of the silver nanoparticles was observed, which the authors suggest as the probable mechanism of tolerance. The studied bacteria did not show any growth reduction in medium containing up to 2.29 mg/L dissolved silver. The authors consider all dissolved silver as silver ions, although it is possible that most of them would precipitate in the form of AgCl or other salts (as Luria Broth, used in this experiment, contains a substantial amount of NaCl—typically 5 g/L).

It was reported that engineered silver-tolerant *Escherichia coli* uses a peptide motif (AgBP2) to bind silver, and nanoparticle-like, electron-dense objects were found in the bacteria exposed to silver ions. The formation of nanoparticles-like objects could be a resistance mechanism, which decreases the bioavailability of silver. Fibrillation of proteins in amyloidogenic diseases is increasingly considered as a protective mechanism to remove toxic oligomeric forms of the proteins, so there is a precedent for such a mechanism of defence [116]. It was shown that some other silver-resistant bacteria (*Pseudomonas stutzeri* AG259, isolated from a silver mine) produce silver crystals of up to 200 nm in size after exposure to 50 mM silver nitrate [113]. It was proposed that the reduction of toxic silver ions into less-reactive metallic particles contributes to *P. stutzeri*’s silver resistance. The peptide motif expressed by engineered, silver-tolerant strain of *E. coli* does not increase the bacterial tolerance against copper ions, so it seems to be silver-specific [113]. The authors suggest that engineered, silver-resistant bacteria could be used in various applications, ranging from remediation to interrogation of biomolecule-metal interactions *in vivo*. As there is a growing concern regarding bacterial resistance to silver (discussed already in previous sections), the idea of creating new mechanisms involved in this phenomenon may be questioned, and cost-benefit analysis should be considered fully.

Although, as mentioned above, there are some studies done on the mechanisms of bacterial resistance to silver (the issue itself is known for a long time), the prevalence of this phenomenon is not yet well understood. Basing on their results, some authors state that in clinical isolates the prevalence of silver resistant strains is low [117,118]. It should be kept in mind that according to many authors bacterial resistance to silver (based on several mechanisms) is encoded on plasmids [119,120,121], which opens an opportunity for transfer of relevant genes between bacterial strains (as happened in the case of antibiotics; reviewed in [122] and others). As resistance to silver has already appeared is several different environments (as described above) it could potentially spread to other bacterial strains. According to Chopra [118], the emergence of resistance can be minimized if the level of silver ions released from products is high and the bactericidal activity rapid (so wound dressings releasing low levels of silver are potentially more dangerous). This author also draws attention to the issue of standardization of antimicrobial testing methods for silver, and added to this there is a critical need for standardized methods to discriminate between nano, ionic and molecular forms of silver, especially in light of emerging data suggesting *in situ* precipitation of silver particles as a defence mechanism.

Bacterial resistance to silver is of significant concern from a societal viewpoint (e.g., references given by Lansdown, [6]; studies of Khan *et al.* [88] and Hall Sedlak *et al.* [113]), because of the serious threat implied for human health in hospitals and other environments, especially for immune suppressed patients and the potential for the advantages offered by nanosilver as an anti-microbial agent to be lost if silver is used widely in consumer products without due consideration of the cost-benefit ratio. In addition, there is a desire to preserve the remaining second line antibiotics for such critical situations, and the use of silver as a generalized antibiotic is therefore of significance. Silver can also be used as an antifungal and antiviral agent (see below). As yet there have only been limited discussions of the potential for silver resistant bacteria to emerge, but this is clearly a matter of some interest in deciding on the future range of applications for silver nanoparticles and their regulation. Issues such as silver nanoparticle activity against antibiotic-resistant bacteria in hospitals, medical devices and other key applications have not been fully evaluated. However, this is a significant issue that may inform future policy decisions on the use and regulation of silver nanoparticles. Some authors [112,123] already drew attention to bacterial resistance to silver. As can be concluded from their work, wide-spread usage of products containing silver components may result in more wide-spread bacterial resistance to those antimicrobial agents, and, as a result, loss of another “defense line” against drug-resistant bacteria

Silver nanoparticles and other silver components have also an impact on bacterial communities. Several studies on silver release and behaviour in WWTPs, in which this impact was investigated, were already mentioned (e.g., [43,59,60]). In the study of Fabrega *et al.* [124] increasing concentrations of silver nanoparticles caused a decrease of biofilm biomass and volume. Also, uptake of silver nanoparticles showed a concentration-dependent pattern. The major bacterial groups were present in biofilms irrespective of silver nanoparticle exposure, but the biofilm succession and the relative abundance of major bacterial groups were impeded in silver nanoparticle-treated biofilms, which may suggest longer term effects on biofilm development and function.

In the study of Das *et al.* [125] carboxy-functionalised silver nanoparticles (at concentrations up to 1 mg/L) were found to cause rapid, but temporal inhibition of bacterioplancton production. The authors observed four responses to silver nanoparticle exposure: (a) intolerant; (b) impacted but recovering; (c) tolerant; (d) stimulated. Bacteria belonging to groups (b) and (c) were responsible for most of the bacterioplancton production observed. Bacteria belonging to group (d) were rare and did not contribute significantly to the overall community’s productivity. As stated by the authors, natural bacterial communities can tolerate a single, low dose of silver nanoparticles. The bacterial community activity level 5 days after the exposure was similar to that of the negative control, but the community composition was changed.

In the study of Colman *et al.* [126] the response of streamwater and sediment microorganisms to commercially available silver nanoparticles was investigated. Silver nitrate was used as a control. As observed by the authors, the short-term biological impacts of silver nanoparticles were attenuated by physical and chemical properties of both sediment and streamwater.

## 8. Cytotoxicity, Genotoxicity, Oxidative Stress, Inflammation in Mammalian Cells

The available data on the impact of silver nanoparticles on mammalian cells was reviewed by Johnston *et al.* [61] in 2010, so here we only summarize the most important points and supplement it with the latest outcomes from the literature.

As pointed out by Johnston *et al.* [61], inflammatory, oxidative, genotoxic, and cytotoxic consequences are associated with silver particulate exposure, and are inherently linked. However, there is still not enough data available to conclude what the toxic effects of silver nanoparticles are, and making correlations between findings is difficult due to the variety of different silver nanoparticles and stabilizing surfaces utilized in various studies, combined with the limited characterization of the nanoparticles dispersion characteristics and stability over the exposure time. Several problems in experimental design and the data presentation were pointed out. The lack of differentiation between particulate and soluble forms of silver, insufficiently described experimental details, no or incomplete toxicological data for *in vivo* distribution studies and excessively high, irrelevant doses. This list can be supplemented with the lack of time-resolved data to enable kinetics of responses to be determined, the lack of correlation between nanoparticle localization and impacts, and the data gap in terms of the impacts of silver nanoparticles on protein and enzyme function, as discussed above.

From *in vivo* studies, the liver is the primary site of silver particulate accumulation, although there is some evidence that silver has very high biopersistence in both brain and testis [127]. Further studies are needed for better understanding of underlying mechanism leading to such a specific localization. Many studies were focused on the tissue distribution of silver nanoparticles, which helps to design relevant *in vitro* experiments (on relevant cell lines, corresponding to potential target sites of toxicity). Johnston *et al.* [61] regard it as being of particular importance, as many kinds of nanomaterials require toxicological assessment within predictive, relevant, *in vitro* models. Based on those conclusions, the use of liver cell lines [79] seems to be justifiable. Other authors state that due to the presence of silver nanoparticles in sprays, the lungs should be considered as the main uptake route [42]. Johnston *et al.* [61] refer to several studies in which this route of exposure is investigated, and conclude that silver nanoparticle toxicity is driven by the exposure time and concentration, the method of particle administration, and particle size (and agglomeration). Among more recent studies, Foldbjerg *et al.* [63] also used a lung-derived cell line as the most relevant one.

According to Johnston *et al.* [61], silver nanoparticles exploitation within textiles and wound dressings enables particles to come into direct contact with skin. Most available studies consider the efficiency of silver-enriched wound dressings at preventing infection and related topics. Human mesenchymal stem cells were used in the study of Hackenberg *et al.* [128]. The choice can be easily justified, as hMSC are an essential player in wound healing and tissue regeneration and they are in close contact with implant surface areas and may be exposed to silver nanoparticle-containing coatings and wound dressings. Primary fibroblasts from mouse were used also by Arora *et al.* [129], as the study was related to the intended use of silver nanoparticles in the form of a topical antimicrobial gel formulation for the treatment of burns and wounds.

An interesting study on the kinetics of the tissue distribution of silver nanoparticles of different sizes was conducted by Lankveld *et al.* [62]. Silver nanoparticles were intravenously administrated to rats. According to their results, accumulation occurs in all organs regardless of particle size (except for 80 nm particles which were not observed in blood). For 80 nm and 110 nm silver nanoparticles most accumulation occurred in the spleen, liver and lungs, while for 20 nm particles accumulation was primarily in the liver, kidneys and spleen. For other organs (brain, heart and testes) particle size did not seem to play an important role. After each injection, silver nanoparticles were rapidly distributed from blood to other tissues. For all time points the concentration of 20 nm silver nanoparticles in liver, spleen and lung was remarkably lower than those of larger nanoparticles, possibly due to faster elimination/excretion, dissolution or redistribution to other organs, not evaluated in the study. An important note is that the concentration of silver in different organs was measured using ICP-MS, which does not allow distinguishing between ionic and nano form. Even if the route of exposure seems to be quite irrelevant from an exposure viewpoint (intravenous administration), the authors explained that it allows investigation of internal exposure (bypassing the exposure issue). Also, Johnston *et al.* [61] states that intravenous administration of nanoparticles has the ability to highlight which organs are most at risk from their toxicity. However, it should be noted that there is increasing evidence that route of exposure does affect final biodistribution, as the initial biomolecules that coat the nanoparticles (e.g., lung surfactant proteins or blood plasma proteins) influence the final distribution. According to other authors, the biodistribution of metallic nanoparticles depends on their size [130], surface charge and coating [131].

Johnston *et al.* [61] also highlight the importance of experimental design and its impact on the outcomes of the conducted studies. For example, excessively high doses (e.g., [132]), unrealistic from a practical point of view, are very commonly used. Another problem is insufficient information on the experimental design (including the nanomaterial characteristics under exposure conditions). As can be concluded from more recent studies, the importance of proper material characterization in the relevant exposure media and over the time-course of the experiment and careful experimental design is becoming better understood.

More and more experimental data are emerging, but due to the variety of materials, biological models and experimental methods used it is very hard to draw any conclusive statements regarding the toxicity mechanisms of silver nanoparticles. Examples of experiments in which different materials and biological models were used, as well as a summary of their results are presented in Appendix for *in vivo* and *in vitro* studies.

In the study of Cao *et al.* [31] Ag-PIII nanoparticle-modified surfaces introduce very little toxicity towards osteoblast-like cell lines. Moreover, an improvement in osteoblast proliferation was found as the effect of Ag-PIII surface modification (compared to standard bone implant surfaces). The authors suggest that the observed effect was due to the presence of proton-depleted regions (between cells and the studied surface). The limited size of the proton depleted regions cannot affect the overall proton electrochemical gradient of a eukaryotic cell and thus does not interfere with the synthesis of ATP. On the contrary, the aggregated protons adjacent to the Ag-PIII surface may promote the overall energy-dependent reactions and proliferation on the macroscopic scale because of the larger size. The authors suggest that formation of proton-depleted regions can explain the antibacterial properties of the Ag-PIII surface.

According to AshaRani *et al.* [83] silver nanoparticles (6–20 nm according to TEM) were taken up by cells (normal human lung fibroblast and human glioblastoma cells) and evenly distributed in cytoplasm and nucleus (as confirmed by TEM) and which would be consistent with a non-endocytotic mechanism of uptake enabling the particles to avoid the endo-lysosomal pathway [133]. Reduced uptake at low temperature suggests an active uptake mechanism. In this study, silver nanoparticles were found to cause several adverse effects: chromosome instability, mitotic arrest, and significant alterations in cell morphology. The toxicity of the silver nanoparticles was found to be mediated by intracellular calcium. Several differences were found between normal and cancerous cells, which once again underline the importance of relevant cellular models for biological studies. Normal human fibroblasts efficiently recovered from mitotic arrest caused by silver nanoparticles, but cancer cells (human glioblastoma) could not recover and thus proliferation was decreased. It is important to notice that the silver concentrations used in these experiments were very high (up to 400 μg/mL).

## 9. Cell Cycle Effects and Link to Reproductive/Developmental Toxicity/Neurotoxicity/Immune and other Less Well Understood Effects

In the study of Braydich-Stolle *et al.* [68] a silver nanoparticle size and coating dependent decline in mouse spermatogonial stem cell (C18-4) proliferation at concentrations ≥10 μg/mL was found. Several sizes of hydrocarbon and polysaccharide-coated silver nanoparticles (10, 15, 25–30, 80 nm) were used in the experiment. Disruption of GDNF/Fyn kinase signalling was found to be the cause of the decreased proliferation of the mouse spermatogonial stem cells. ROS production and/or apoptosis did not seem to play a major role. Silver nanoparticles were localized in cytoplasm and in lysosomes (confirmed by TEM and confocal microscopy). The coating of at least some was degraded upon interaction with the intracellular microenvironment (confirmed after the incubation in ALF for 72 h, which caused increased agglomeration due to coating degradation), reducing the biocompatibility of the silver nanoparticles, as observed by a time-dependent onset of toxicity. The effect of media supernatant that had been pre-treated for 24 or 72 h with the silver nanoparticles (which were then removed by sedimentation) on the C18-4 cells was negligible, so the authors concluded that silver ions did not have much impact on studied cells, although it may be that the intact coating prevented silver ion dissolution (the content of dissolved silver has not been assessed). It was also shown that silver nanoparticles are able to significantly decrease the activity of a purified commercially available Fyn kinase, which plays an important role in the GDNF-induced signalling cascade in the C18-4 cells. The very low concentrations at which effects were observed (≥10 μg/mL) make these results more relevant to environmental exposure conditions. The observed impact of silver on spermatogonial stem cells also raises a question of possible adverse effects of silver nanoparticles on reproductive and developmental processes.

In the study of Austin *et al.* [23], two types of peptide-conjugated silver nanoparticles were used—RGD-silver nanoparticles and NLS/RGD-silver nanoparticles. All silver nanoparticles were spherical, 35 nm in size. Nuclear targeting signal (NLS) is a nuclear tag which can be bound in cytoplasm and is actively transported into the nucleus through the nuclear pore complex. Arginine-glycine-aspartic acid peptide (RDG) is known to assist in receptor-mediated endocytosis in cancer cells. Silver nanoparticles conjugated with NLS were localized at the nucleus of both cell models (human oral squamous cells carcinoma (HSC-3) and human keratinocytes (HaCat) cells), while RGD-AgNPs and PEG-AgNPs were dispersed within the cytoplasm. NLS/RGD-silver nanoparticles caused dose-dependent DNA double-strand breaks (starting from 0.1 nM, comparing to 0.4 nM for NLS/RDG-gold nanoparticles) and a subsequent increase in the sub G1 (apoptotic) population in the cancer cell model, which the authors consider as an evidence of their harmful, toxic effects. The observed increase in apoptotic populations is only induced when silver nanoparticles are localized at the nucleus. In addition, an accumulation of cells in the G2/M phase of the cell cycle was observed in both cell models when treated with the NLS-functionalized silver nanoparticles, which indicates that the presence of NSL-silver nanoparticles disrupted the transition of G2/M or M/G1. Complete cell cycle analysis and cell cycle synchronization experiments showed that the arrest takes place in the G2 phase, which indicates that the presence of NLS-silver nanoparticles prevents normal transitions between the different phases of the cell cycle. In comparison, cancerous and healthy cells treated with RGD-silver nanoparticles did not show a significant increase in the sub G1 population when compared to their controls but did show G2/M accumulation. It is worth noticing that the authors use low doses of silver nanoparticles (0.1 nM and 0.4 nM). From their experimental results, the authors consider ROS generation as the main cause of the cell cycle alteration observed. The impact of ROS on cell cycle progression has been known for several years According to Boonstra *et al.* [134] , the impact of ROS on cell cycle depends on the amount and duration of ROS exposure. Low levels of ROS activate growth factor stimulated signalling cascades, which results in increased cell cycle progression. On the other hand, prolonged exposure to ROS results in differentiation-like growth arrest. Additionally, the ability of nanoparticles (including combustion particles) to induce ROS is also well established [135]. Indeed a recent paper suggests that a nanomaterials’ conduction band energy levels (band gap) and particle dissolution could be the basis of predicting its oxidative stress potential and subsequent pulmonary inflammation potential as the basis of a predictive toxicity platform [136].

The impact of stable, non-aggregated silver nanoparticles (11.6 ± 3.5 nm in size) on zebrafish embryo development was observed by Lee *et al.* [65]. Silver nanoparticles were found to passively diffuse into developing embryos via chorion pore canals. The authors found that both toxicity and the types of abnormalities in zebrafish embryo are highly dependent on the silver nanoparticles dose. As both normal and deformed zebrafish developed from embryos exposed to the same suspension of silver nanoparticles, the authors conclude that some embryos may be inherently more tolerant than others. The authors compared observed abnormalities to those caused by other tested chemicals and found that the effects of silver nanoparticles are similar to those of cadmium, dichloroacetic acid and 2,3,7,8-tetrachlorodibenzo-p-dioxin.

There is very little information on inflammatory responses caused by silver nanoparticles. Yen *et al.* [76] found that silver nanoparticles do not cause proinflammatory genes (TNF α, IL-1, IL-6) upregulation in J774 A1 macrophages, but gold nanoparticles of similar size show proinflammatory potential. As mentioned previously, the authors hypothesize that the observed differences were caused by different surface charge and uptake pathway. In the study of Trickler *et al.* [77] the interactions between silver nanoparticles (three sizes: 28.3 ± 9.6 nm, 47.5 ± 5.6 nm, 102.2 ± 32.8 nm) and primary rat brain microvessel endothelial cells (rBMEC) were investigated. Cytotoxic responses were found to be size-dependent (smaller nanoparticles caused cytotoxic responses at lower doses than larger ones) and proinflammatory responses (IL-1β, TNF α and PGE 2 release) showed both size and time-dependent profiles. The time dependence may also be related to particle uptake rates / amounts, which are known to be size dependent. Blood-brain barrier permeability also showed a size-dependent increase (smaller nanoparticles were more likely to pass the barrier), which probably correlated with increased immunotoxicity. The authors conclude that their study results suggest a proinflammatory and neurotoxic potential of silver nanoparticles. An aspect of this study which is of particular value is the assessment of kinetics of proinflammatory response. Generally, time-resolved studies on cytotoxicity, genotoxicity, immunotoxicity are lacking.

## 10. Disentangling Impacts from Silver Ions versus Silver Nanoparticles 

**Figure 4:**
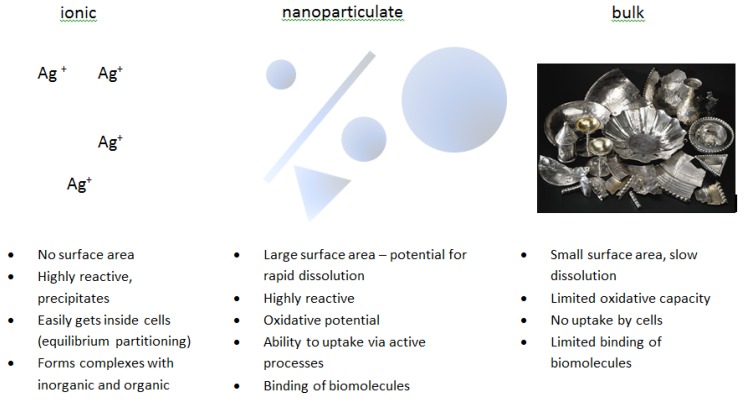
Main differences between ionic, nanoparticulate and bulk silver.

One of the crucial issues in all biological studies on the environmental or biological impacts of silver nanoparticles is how to distinguish the impact of nanoparticles and their specific characteristics (e.g., size, shape, coating) from the impact of silver ions (and other forms of silver) present in the solution. Although in some studies there is no control allowing to distinguish between silver ions and silver nanoparticle impacts [77,128], in most cases the need for additional data on silver ions impacts as a control is well recognized, although the optimal method to include silver ions controls is not well established, due to differences in uptake mechanisms between the different forms *etc.* Summary of differences between ionic, nanoparticulate and bulk silver is presented on Figure 4.

### 10.1. Particle Uptake and Localization Mechanisms

Where studies have confirmed the nanoparticulate nature of the silver, information on the localization is also vital to help interpret the data. Some authors confirmed localization in the lysosomes (e.g., [68]), several studies mentioned silver nanoparticles localized in nuclei and resultant DNA damage which suggests a direct interaction rather than an indirect DNA damage as a late-stage of apoptosis (e.g., [23]).

### 10.2. Silver Ions *versus* Silver Nanoparticles

The most common strategy is to use, as a control, an equivalent mass of silver in the form of AgNO_3_ (or another soluble silver salt) [91]. If the same medium is used for control and silver nanoparticle samples, the amount of precipitated insoluble salts should be the same. In any case, it is important to remember that the concentration of inorganic salts in commonly used media is usually higher than in the natural environment. Note also that the OECD reference media for different species used in ecotoxicological studies (daphnia, algae, *etc.*) are significantly different and dispersions also can be significantly different in those media [137], again leading to significant problems of comparing data and assessing their relevance to real exposures. The OECD should address this issue in their review of the applicability of existing toxicological tests to nanoparticles.

In the study of Suresh *et al.* [91] the AgNO_3_ IC50 values for RAW macrophages and lung epithelial cells were 0.75 and 1.8 μg/mL respectively. Even if high concentrations of silver nanoparticles were used (much higher than the inhibitory concentrations), the estimated concentration of dissolved silver ions was several fold below that required to induce cytotoxicity. Analysing available dissolution data, the authors concluded that the relative differences in cytotoxicity for the various silver nanoparticles examined was not due to the liberation of silver ions into the medium. Although the role of silver ions in nanoparticle toxicity is acknowledged, the authors state that it is rather the influence of surface coatings on *in vivo* dissolution and increased localized concentration of Ag ions in locations that ions would not normally reach that may be important and not the bulk silver ion concentrations that are typically measured.

In many studies, silver ions (in the form of AgNO_3_) caused similar effects to those induced by silver nanoparticles, although silver ions were active at lower concentrations. In the study of Foldbjerg *et al.* [63] EC50 values for silver ions were two times lower than those of silver nanoparticles. On the other hand, silver nanoparticles were found to generate more ROS than silver ions (e.g., equation proposed by Liu *et al.* [94]), which indicates that the ROS production is due to specific characteristics of silver nanoparticles, not only due to ion release. It could be argued that it is not the presence of silver ions, but the process of their release, that generates ROS. According to the authors, the reduction of toxicity of both silver nanoparticles and silver ions after the addition of N-acetyl-L-cysteine proved the involvement of ROS (a strong correlation between the levels of reactive oxygen species (ROS) and mitochondrial damage or early apoptosis was observed) in the observed toxicity. N-acetyl-L-cysteine is an antioxidant, but it can also bind silver ions to its sulfhydryl groups, so in the case of silver ions strong silver-sulphur(II) binding [55] could prevent any impact, not necessarily by neutralization of ROS.

$nAg+O_{2}\to nAg\cdots O_{2}\overset{H^{+}}{\to }Ag^{+}+reactive\;oxigen\;intemediates+nAg\overset{H^{+}}{\to }Ag^{+}+H_{2}O$ [94]

In the study of Bouwmeester *et al.* [66] the passage of silver nanoparticles (four sizes, 20 to 113 nm according to TEM analysis) and silver ions through a co-culture of Caco-2 and M cells, and their effects on whole-genome mRNA expression were investigated. The nanoparticles used in the study were carefully characterized. The rate of silver ions release was found to be 6%–17% after 24 h incubation in cell culture medium (DMEM or RPMI 1640, both supplemented with non-essential amino acids and 10% of heat-inactivated fetal bovine serum), depending on nanoparticle size. Only quite high dose (37.5 μg/mL) of smallest studied nanoparticles (20 nm) had an effect on cell viability. In contrast, even low doses (2.5 μg/mL) of AgNO_3_ significantly decreased number of viable Caco-2 cells. Exposure to silver nanoparticles did not affect the monolayer integrity, and the amount of silver translocated through the barrier was the same for silver nanoparticles and AgNO_3_. Expression of several genes was altered due to the exposure to silver nanoparticles, although no nanoparticle-specific effects were observed (AgNO_3_ induced expression of the same genes). Based on their results, the authors exclude a “Trojan horse” mechanism of gene expression alterations (in such a case the impact from silver nanoparticles would be stronger than that of AgNO_3_) and conclude that the observed effects were solely due to silver ion exposure.

In undifferentiated PC12 (derived from a pheochromocytoma of the rat adrenal medulla) cells, citrate-coated silver nanoparticles (average size 6 nm) impaired DNA synthesis, but to a lesser extent than an equivalent nominal concentration of silver ions (which is in agreement with other studies) [64]. Silver nanoparticles and silver ions were equally effective against protein synthesis. In differentiating cells, silver nanoparticles caused oxidative stress and impaired cellular differentiation. The effects of silver nanoparticles cannot be explained solely by silver ions release, and are dependent on nanoparticle size and coating (which, in turn, affects their solubility). As silica nanoparticles showed no impact, the observed effects seemed to be material-specific. As significant differences in potencies and differentiation outcomes were found for citrate-coated and PVP-coated silver nanoparticles, both particle size and coating seemed to play a role in the impact of silver nanoparticles on the studied cells. The authors point out several differences between the interactions of silver nanoparticles and silver ions that could explain their differential effects on studied cells:

- The lack of selectivity of citrate-coated silver nanoparticles toward DNA versus protein synthesis in undifferentiated cells, whereas silver ion is highly selective towards DNA.
- The inability of ascorbate (antioxidant) to protect cells from oxidative stress and cell loss caused by citrate-coated silver nanoparticles, whereas the same antioxidant is protective against silver ions [138], which implies that cell loss from citrate-coated silver nanoparticles reflects a different underlying mechanism and that, for the nanomaterial, oxidative stress is a result of cytotoxicity, not a cause of it.
- The greater inhibition of protein synthesis at lower concentrations of citrate-coated silver nanoparticles and a loss of effect at higher concentrations, totally distinct from the monotonic dose–effect relationship for silver ions [138] which indicates that low concentrations of citrate-coated silver nanoparticles disrupt protein synthesis through a mechanism unrelated to freely dissolved silver ions.
- The limited effectiveness of citrate-coated silver nanoparticles at suppressing the acetylcholine phenotype whereas silver ions affect both acetylcholine and dopamine phenotypes.

In the study of Austin *et al.* [23], NLS-silver nanoparticles were found to cause cell-cycle arrest in cancer cells. No such effect was found after treatment with AgNO_3_, and cell cycle arrest was, according to the authors, caused by ROS generated by silver nanoparticles.

In the already mentioned study of Liu *et al.* [94] elements of the drug delivery paradigm were applied to silver nanoparticle dissolution (another part of the study, chemical concepts for controlled release, were presented in an earlier section of this review). As suggested by the authors, silver nanoparticles behave in analogy to a drug delivery system, in which the particle contains a concentrated inventory of an active species, the ion, which is transported to and released near the biological target sites.

## 11. Conclusions

Despite its long history of use in consumer products, the mechanisms of action of colloidal and nanoscale silver particles in the environment and for humans have yet to be fully elucidated. There are several unsolved problems, which appear in most (or many) published studies. The most important ones seem to be a reliable method of assessment of silver nanoparticle uptake and proper characterization and quantification of nanoparticle load in relevant medium and *in situ* (inside cells/organisms) in a time-resolved manner. Lack of such assessments probably explains (at least partly) differences between experimental results and difficulties in toxicological data interpretation. We can state then that a proper connection between analytical techniques and toxicological studies has yet to be established.

An on-going challenge is the need to distinguish between effects from dissolved ionic silver versus nanoparticulate silver, despite the fact that the mechanisms and sites of action of the two are extremely different: ionic silver diffuses across biological barriers and reaches equilibrium concentrations throughout most organs/cells (although with evidence of biopersistence in the brain), whereas nanoscale silver particles maybe actively taken up with the potential for high accumulation in specifically cellular/sub-cellular locations, whereupon subsequent dissolution would release unnaturally high local ionic concentrations with significant potential for new, unforeseen consequences.

A drawback in most of the published *in vitro* studies is the fact that only one time-point (typically 24 h) is studied. This has three significant consequences: transitory effects that cells can recovery from quickly are missed; effects that may take longer to manifest (e.g., signalling from cells/sub-cellular organelles that have taken up nanoparticles) may be missed completely if they have not yet occurred or are below detection levels still; and the sequence of events cannot be teased out where more than one end-point is assessed. Additionally, the lack of uptake mechanism and localization data makes interpreting silver toxicity data difficult, especially in light of the fact that ionic and particulate forms may behave quite differently.

Coupled to this is the lack of appreciation for the fact that nanoparticles are generally quite intrinsically unstable, which means that they are evolving (ageing), with significant consequences for their biological and environmental impacts as this can affect the ratio of particulate nanosilver to ionic silver. Thus, it is clear that literature reports on silver nanoparticle toxicity should also make some comment (where possible) on the “age” of their samples at the time of study, or include a simulated “ageing” experiment. A possible mechanism for mitigating the effects of silver nanoparticle dissolution during storage (ageing) could be the removal of ionic silver from the suspensions just before the experiment, e.g., by column purification approaches. To prevent silver nanoparticles dissolution they can be stored under anoxic conditions, for example in suspension purged with Ar or N_2_.

The potential for bacterial resistance to silver (in any of its forms) seems to be of the greatest concern. Although several studies are available (some of them cited and discussed in this review), the mechanism of the appearance and spread of bacterial resistance to silver (ions and particles) is not completely clear. Despite this, we can presume that wide-spread and uncontrolled usage of products containing silver nanoforms may lead to a growing severity of this problem. Generally, the proven bacterial resistance to silver and the negative impact of silver on natural bacterial communities (and water organisms) are the most important issues which should be taken into account in regulatory processes, and indeed deciphering the sources of effect as being from ionic silver, nanosilver or indeed a combination of both will be vital in terms of undertaking a risk—benefit analysis of different potential applications. There is an urgent need for studies concerning mechanisms, prevalence and epidemiology of bacterial resistance to silver, as this information is still lacking.

A final comment for the planning of new studies to address some of the knowledge gaps identified here is the fact that a fuller description of the nanoparticles themselves is required, especially as they exist, and age, in the exposure medium over the whole time-course of the exposure studies. This includes a description of any coating or dispersing entities, as well as an assessment of the particle (and coating) stability in the exposure media and following uptake and localization. Thus, coupling kinetics of uptake to localization and to kinetics of particle degradation/dissolution and kinetics of biological impact is extremely important to truly tease-out mechanisms of interaction and modes of action, especially where there can be combined impacts from nanoscale particles and dissolved ionic species, as in the case of silver nanoparticles.

Without a concerted and agreed effort to raise the quality of studies being performed and reported, in general in the nanotoxicity area, and specifically for nanosilver, it is hard to see how the field can progress beyond its current state, whereby studies exist in isolation and are not comparable to one another. Complete experimental descriptions, including characterization in a time-resolved manner would allow cross-comparison between particles and experimental systems, and could in time lead to the development of quantitative structure-activity or structure-property relationships as the basis for predictive toxicology. On the basis of currently available data, no such comparisons or predictions could be achieved as the characterization and time-resolved data is simply not available.

## Abbreviations

Ag-PIII: Single Step Silver Plasma Immersion Ion Implantation
ALF: Artificial Lysosomal Fluid
ATP: Adenosine-5′-triphosphate
CEN: Comité Européen de Normalisation (European Committee for Standardization)
CVC: Central Venous Catheter
DLS: Dynamic Light Scattering
DLVO: Dejaguin–Landau–Verwey–Overbeek (theory)
DMEM: Dulbecco’s Modified Eagle Medium
EDTA: Ethylenediaminetetraacetic acid
EDX: Energy-dispersive X-ray spectroscopy
EFSA: European Food Safety Authority
EPS: Exopolysaccharides, extracellular polymeric substances
GIT: Gastrointestinal tract
HSA: Human Serum Albumin
ISO: International Organization for Standardization
ISO/TS: International Organization for Standardization/Technical Specification
ICP-MS: Inductively coupled plasma mass spectrometry
OECD: Organisation for Economic Co-operation and Development
PVP: Polyvinylpyrrolidone
ROS: Reactive Oxygen Species
RPMI: Roswell Park Memorial Institute medium
SEM: Scanning Electron Microscopy
TEM: Transmission Electron Microscopy
USEPA: The United States Environmental Protection Agency
WWTP: Wastewater Treatment Plant

## Appendices

**Table A1:** Transformation of silver nanoparticles in the environment.

Study	Material	Environment	Kind of transformation	Factors influencing transformations
Kittler *et al.* [69]	Citrate and PVP-coated 85 nm silver nanoparticles	Water	dissolution (over 125 days)	some nanoparticles released up to 90% of their weight into water, but the dissolution was never complete the final degree of dissolution did not depend on the absolute concentration of nanoparticles, but seemed to be characteristic for certain nanoparticles the rate of dissolution was found to be temperature-dependent the dissolution rate was higher for PVP-stabilised nanoparticles than for citrate-stabilised nanoparticles, probably due to reductive properties of citrate time-dependent dissolution leading to higher toxicity
Liu *et al.* [93]	citrate-stabilised, small (4.8 ± 1.6 nm) silver nanoparticles	distilled water acetate buffers (5 mM, pH 4,5 and 5.6) and borate buffers (5 mM, pH 7.4, 8, and 9) seawater and water supplemented with NOM	dissolution	higher temperature (in 0–37°C range)→higher silver ions release lower pH→higher silver ions release addition of organic matter→lower silver ions release absorption of silver ions onto the surface of nanoparticles leads to depletion of silver ions from the solution silver nanoparticles are not persistent in environments containing oxygen
Liu *et al.* [94]	silver nanoparticles microscopic silver foils	Biological medium of different NaCl concentrations	Dissolution Insoluble/low soluble silver salts formation	thiol and citrate ligand binding, formation of sulfidic coatings, and the scavenging of peroxy-intermediates slowed the silver ion release. peroxidation or particle size reduction accelerate dissolution the silver ion release profile could be modified by polymer coatings with complexation sites. silver ion concentration dropped rapidly with increasing chloride concentration, but further increase of chloride content (beyond 2–3 mM) caused an increase of AgCl*_x_*^1−*x*^, which enhanced the amount of total dissolved silver.
Akaighe *et al.* [99]	Silver nanoparticles	Medium containing Suwanee River humic acids, sedimentary humic acids and humic acids from soil samples	Formation of new nanoparticles near the parent ones	Presence and type of humic acids Higher temperature accelerates formation of new particles
Chappell *et al.* [81]	Concentrated silver nanoparticles 30–50 nm in size	1 mM NaNO_3_ 100 mM NaNO_3_	Dispersion stability, dissolution Stabilization by different dispersants	non-ionic surfactant (BRIJ 35) provided good dispersion of silver nanoparticles (unaffected by electrolyte concentration), EDTA and alginic acid did not work well as chemical dispersants (surfactants) polymer loading may enhance the dissolution and release of dissolved silver into the environment sorption of hydrophilic groups may slow down agglomeration of nanoparticles more hydrophobic compounds may promote destabilization of the dispersion
Glover *et al.* [10]	Silver nanoparticles of different size and coating Bulk silver objects	Air (humidity from 0 to >50%)	Formation of new nanoparticles near the parent ones	relative air humidity (no new nanoparticles formation in 0% humidity) exposure to light
Khan 45 [58]	Silver nanoparticles around 65 nm in size	Water Medium containing bacterial exopolysaccharides (EPS)	Stabilization (organic compounds) agglomeration	ESP presence alters surface charge of nanoparticles and reduces agglomeration EPS adsorption was dependent on pH, salt concentration and EPS concentration
Stebounova *et al.* [82]	polymer-coated silver NPs; silver NPs with unspecified coating	two simulated biological fluids representative of the fluids present in lungs	aggregation sedimentation	Initial concentration of nanoparticles Surface charge (higher surface charge → higher stability in suspension Lower pH → lower stability
Unrine *et al.* [97]	gum arabic coated silver nanoparticles PVP coated silver nanoparticles	surface water; water and sediment; water and aquatic plants; water, sediment, and aquatic plants	dissolution aggregation	dissolved organic matter (DOM) bound silver ions DOM stabilized PVP-coated silver nanoparticles as primary particles DOM caused the removal of gum arabic coated nanoparticles from water, possibly due to dissolution and binding of released silver ions

**Table A2:** Silver nanoparticles used in *in vivo* biological experiments and their impacts.

Study	Shape	Size (nm)	Coating	IC50	Organism	Main outcomes
Lee *et al.* 2007 [65]	spherical	11.6 ± 3.5	citrate	~0.3 nM	Danio rerio (zebra fish), embryos	Silver nanoparticles passively diffuse into developing embryos via chorion pore canals both biocompatibility and the types of abnormalities in zebrafish are highly dependent on the silver nanoparticles dose
Rahman *et al.* 2009 [132]	Mainly spherical	25	Not stated (negatively charged)	Not provided	C57BL/6N mice	Silver nanoparticles alternated genes expression the expression in the caudate nucleus, frontal cortex and hippocampus neurotoxicity caused by free radical-induced oxidative stress and by altering gene expression, leading to apoptosis
Lankveld *et al.* 2010 [62]	Not stated	20, 80, 110	Not stated	Not provided	Wistar rats	Silver nanoparticles disappeared rapidly from the blood and were distributed to all organs evaluated (liver, lungs, spleen, brain, heart, kidneys and testes) Size-dependent tissue distribution suggests size-dependent toxicity and health risks. Repeated administration resulted in accumulation in liver, lung and spleen
Lee *et al.* 2012 [70]	spherical	60–100 (powder type), 10 (citrate stabilized)	Citrate, not stated for powder type	EC50 (48h): powder-type: 0.75μg/L total Ag and 0.37μg/L dissolved Ag; Citrate stabilised AgNPs: 7.98μg/L total Ag and 0.88 μg/L dissolved Ag.	Daphnia magna	Silver nanoparticles were acutely toxic to D. magna (due to silver ions release)
Yang *et al.* 2012 [78]	spherical	5, 8, 17, 22, 38, additional large and small	Citrate, PVP, gum arabic	0.6–463 μM range depending on coating and medium (EC_50_—50% growth inhibition)	C. elegans	Toxicity depending on medium ionic strength, both dissolved silver and coating influenced nanoparticles toxicity Linear correlation between silver nanoparticles toxicity and dissolved silver, but no correlation between size and toxicity Some less soluble silver nanoparticles also acted via oxidative stress

**Table A3:** Silver nanoparticles used in *in vivo* biological experiments and their impacts.

Study	Shape	Size (nm)	Coating	IC50	Cell line	Main outcomes
Arora *et al.* 2009 [129]	spherical	7–20	Not stated	61 μg/mL (fibroblasts) and 449 μg/mL (liver cells)	primary fibroblasts and primary liver cells from Swiss albino mice	Silver nanoparticles present in mitochondria and cytoplasm Silver nanoparticles trigger cellular antioxidant mechanisms
Braydich-Stolle *et al.* 2010 [68]	spherical	10, 15, 25–30, 80 nm	Hydrocarbon, polysaccharide	Not provided	mouse spermatogonial stem cells	Size and coating dependent decline in cells proliferation at concentrations ≥10 μg/mL (via disruption of GDNF/Fyn kinase signalling) ROS production and/or apoptosis did not seem to play a major role particle coating was degraded upon interaction with the intracellular microenvironment, reducing biocompatibility small-sized nanoparticles (10–25 nm) are more likely to promote apoptosis or the production of ROS
Trickler *et al.* 2010 [77]	spherical	28.3 ± 9.6, 47.5 ± 5.6, 102.2 ± 32.8	PVP	Not assessed (above concentrations used)	primary rat brain microvessel endothelial cells (rBMEC)	Cytotoxic - size-dependent proinflammatory responses (IL-1β, TNF α and PGE 2 release) - size and time-dependent profiles Blood-brain barrier permeability - size-dependent increase, probably correlated with increased immunotoxicity.
Bouwmeester *et al.* 2011 [66]	spherical	20 ± 2, 34 ± 3, 61 ± 5, 113 ± 8	Not stated	Not provided, but below concentrations studied (5 μg/mL)	Caco-2 and M-cells co-culture	6%–17% of the silver nanoparticles were dissolved to ions The amount of silver ions that passed the Caco-2 cell barrier was equal for the silver ion and nanoparticle exposures Silver nanoparticles caused changes in gene expression in a range of stress responses including oxidative stress, endoplasmatic stress response, and apoptosis observed effects of the silver nanoparticles are likely exerted by the silver ions that are released from the nanoparticles
[31]	Ag NPs embedded in titanium	AgNPs – 5–8	–	no toxic effect	osteoblast-like cell line MG63	–
Foldbjerg *et al.* 2011 [63]	–	60–70 (depending on method) 149 ± 37 in medium	PVP	~5 μg/mL	A549	strong correlation between the levels of ROS and mitochondrial damage and early apoptosis DNA damage induced by ROS
Haase *et al.* 2011 [102]	spherical	20; 40	Small peptide	(μg/mL) 24 h–110; 140 48 h–18; 30	THP-1 cells	significant oxidative stress insides macrophages after exposure to 20 nm silver NPs strongest effects in a concentration range of 20–30 μg/mL silver NP
Hackenberg *et al.* 2011 [128]	Mainly spherical	46 ± 21	Not stated (The mean diameter of NP aggregates was 404 nm)	significant cytotoxicity at 10 μg/mL	human mesenchymal stem cells	Silver nanoparticles found also in nucleus DNA damage, significant increase of IL-6, IL-8 and VEGF release. Migration ability not impaired genotoxic effects at different end points at a subtoxic concentration of 0.1 μg/mL
Powers *et al.* 2010 [138]	Mainly spherical	Citrate – 6, PVP-21 and 75	Citrate, PVP	Not provided	PC12 cell line	Silver nanoparticles exposure impairs neurodevelopment in PC12 cells Effects dependent on cells state of differentiation
Haase *et al.* 2012 [67]	spherical	20, 40	Peptide	Not provided due to data complexity	Mixed primary cell model (mainly neurons and astrocytes) from Wistar rats	Strong size-dependent cytotoxicity of silver nanoparticles further differentiated cultures more sensitive to silver nanoparticles treatment strong oxidative stress responses; acute calcium response preceded oxidative stress responses (and could not be alleviated by antioxidants) Astrocytes more sensitive to silver nanoparticles treatment then neurons Higher uptake of silver nanoparticles by astrocytes (compared to neurons)
Kermanizadeh *et al.* 2012 [79]	Mainly euhedral; elongated or sub-spherical	<20	polyoxylaurat Tween-20	LC50 between 1.25 and 5 mg/cm^2^ (depending on method used)	Liver C3A cells	Greatest level of cytotoxicity comparing to other studied materials Increased IL-8 production, no significant change in TNF-a, IL-6 or CRP was detected
Suresh *et al.* 2012 [91]	spherical	TEM <10, DLS in medium 77–164	poly(diallyldimethylammonium) chloride-Ag, biogenic-Ag, uncoated Ag and oleate-Ag	(μg/mL) 0.1/0.45 0.125/0.7 4.9/6.3 1.1/1.6	mice macrophage RAW-264.7/mice lung epithelial C-10	Cytotoxicity dependent on surface charge and coating, materials used in the synthesis, particle aggregation, and the cell-type lung epithelial cells were found to be more resistant to the silver nanoparticles than the macrophage cells, regardless of the surface coating Toxicity: PDADMAC-Ag > biogenic-Ag and oleate-Ag > colloidal-Ag Membrane damage in response to silver nanoparticle exposure
